# The Effectiveness of Proprioceptive Training for Improving Motor Performance and Motor Dysfunction: A Systematic Review

**DOI:** 10.3389/fresc.2022.830166

**Published:** 2022-04-08

**Authors:** Leoni Winter, Qiyin Huang, Jacquelyn V. L. Sertic, Jürgen Konczak

**Affiliations:** ^1^Human Sensorimotor Control Laboratory, School of Kinesiology, University of Minnesota, Minneapolis, MN, United States; ^2^Center for Clinical Movement Science, University of Minnesota, Minneapolis, MN, United States

**Keywords:** human, motor learning, neurological, orthopedic, proprioception, rehabilitation, sensorimotor, somatosensory

## Abstract

**Objective:**

Proprioceptive training is any intervention aiming to improve proprioceptive function with the ultimate goal to enhance motor function and performance. It has been promoted as an approach to enhance athletic performance and as a tool for sensorimotor rehabilitation. Numerous studies sought to provide evidence on the effectiveness of the approach. However, many different training regimes claiming to train proprioception report a variety of sensorimotor measures that are not directly comparable. This, in turn, makes it difficult to assess effectiveness across approaches. It is the objective of this study to systematically review recent empirical evidence to gain an understanding of which outcome measures are most sensitive, which populations may benefit most from proprioceptive training, and what are the effects on proprioceptive and motor systems.

**Methods:**

Four major databases were searched. The following inclusion criteria were applied: (1) A quantified pre- and post-treatment measure of proprioceptive function. (2) An intervention or training program believed to influence or enhance proprioceptive function. (3) Contained at least one form of treatment or outcome measure that is indicative of somatosensory function and not confounded by information from other sensory modalities. 4) The study reported of at least one quantified measure of motor performance.

**Results:**

Of the 3,297 articles identified by the database search, 70 studies met the inclusion criteria and were included for further review. Across studies, proprioceptive training led to comparable gains in both proprioceptive (+46%) and motor performance (+45%). The majority of studies (50/70) applied active movement interventions. Interventions applying somatosensory stimulation were most successful in clinical populations. Joint position sense error (JPSE) was the most commonly used proprioceptive measure and presents a reliable and feasible measure for clinical use.

**Conclusion:**

Proprioceptive training can lead to significant improvements in proprioceptive and motor function across a range healthy and clinical populations. Regimens requiring active movement of the trainee tended to be most successful in improving sensorimotor performance. Conclusive evidence on how long training gains are retained is still lacking. There is no solid evidence about the underlying long-term neuroplastic changes associated proprioceptive training.

## Introduction

In 2015, our group published a systematic review that summarized the state of knowledge on proprioceptive training at that time (1). Since then, there has been increasing interest into this approach, especially how it affects motor performance in clinical populations and athletes, and what type of interventions may be most suitable and effective for a given population. Here we present a follow-up review that presents a comprehensive overview of the research in this area since 2013.

Broadly defined, proprioception refers to the sense of body position and motion. In addition to position and motion senses, it includes sense of effort, force and heaviness. Proprioceptive training is an intervention that aims to improve proprioceptive function with the ultimate goal of improving or restoring sensorimotor function (1). There is increasing empirical evidence documenting that a training focusing on improving specific aspects of proprioception (e.g., position sense) improves the trained motor function and may also transfer to motor tasks that were not trained (2–5). Conversely, it is now established that motor learning enhances not only a trained motor skill but also proprioceptive function and that it affects neural processing in motor as well as in somatosensory cortical areas (6). It is this close association between motor and proprioceptive learning that is behind the motivation to examine if and what forms of proprioceptive training yield meaningful gains in proprioceptive and motor function, and to identify populations most responsive to such interventions.

Because proprioception and movement are closely linked, and because the motor system uses input from multiple sensory modalities to control movement, it is typically difficult to isolate the contribution of a specific sensory system to observable gains in motor function. Thus, in order to elucidate the extent to which proprioceptive training enhances sensorimotor function, it is important to obtain measures of proprioceptive as well as motor function. Moreover, proprioception can only be accurately quantified during motor tasks that do not rely on other sensory input. For example, postural control requires the integration of visual, vestibular, and proprioceptive information (7). Consequently, when assessing balance, it is difficult to determine the contribution of proprioceptive information during static or dynamic balance without blocking non-proprioceptive signals. For the purpose of this review, we did not include studies that stated to have conducted a proprioceptive training, but only reported biomechanical measures of motor performance to infer indirectly on proprioceptive status. Direct measures of proprioceptive performance included specific somatosensory or somatosensory-motor measures, such as passive or active joint position sense.

This systematic review focused on research produced in the last 7 years (2013–2020) that was published after the previous review by Aman et al. (1). Specifically, we aimed (a) to document interventions that are used to improve proprioception and motor performance, (b) to highlight the measures to quantify the effects on proprioceptive and motor performance due to proprioceptive training, and (c) to examine the usefulness of proprioceptive training as a rehabilitation tool to improve motor function and performance in clinical and non-clinical populations.

## Methods

The methods applied for this review follow the Preferred Reporting Items for Systematic Reviews and Meta-Analysis Literature Search Extension (PRISMA-S) checklist (8).

### Literature Search Strategy

A systematic search of the literature was performed using the databases of Medline (Ovid), CINAHL, PsycInfo (Ovid), and Scopus. The specific search terms were propriocept^*^, kinesthesis, train^*^, rehabilitat^*^, along with other search words including *intervention, therapy, treatment, exercise, learning, human*. An additional search term “*proprioception.mp”* was used in Medline to limit the search to publications that included the term *proprioception* in the title or keywords section. Without this limitation, Medline identified a large number of papers that had no focus on proprioception or proprioceptive training. A full list of the exact combination of search terms used in each database can be found in Appendix A, Supplementary Material.

Each search was limited to languages in English, German, and Chinese because our research team had fluency in these three languages. The search was limited to publication dates from January 2013 to October 20, 2020. The Medline search was further constrained by including the terms *all clinical trials, comparative study, evaluation study, multicenter study, observational study*, or *validation study*. The search in CINAHL was limited to *research article* or *peer reviewed*. Only human studies were evaluated. The year 2013 was chosen, because 2012 was the last year evaluated in the systematic search by Aman et al. (1).

### Inclusion/Exclusion Criteria

The following four inclusion criteria were employed to identify relevant studies: (1) An intervention or training program of any variable length or duration believed to influence proprioception was implemented. (2) A quantified pre- and post-treatment measure of proprioceptive function was reported. (3) Contained at least one form of outcome measure that relies on or is indicative of somatosensory function and is not influenced by information from other sensory modalities (e.g., visual or vestibular). (4) A quantified pre- and post-treatment measurement of motor performance was reported. The first three inclusion criteria duplicate those from Aman et al. (1) and the fourth was subsequently applied to identify studies incorporating motor performance.

### Data Extraction and Reporting

**Figure 1:**
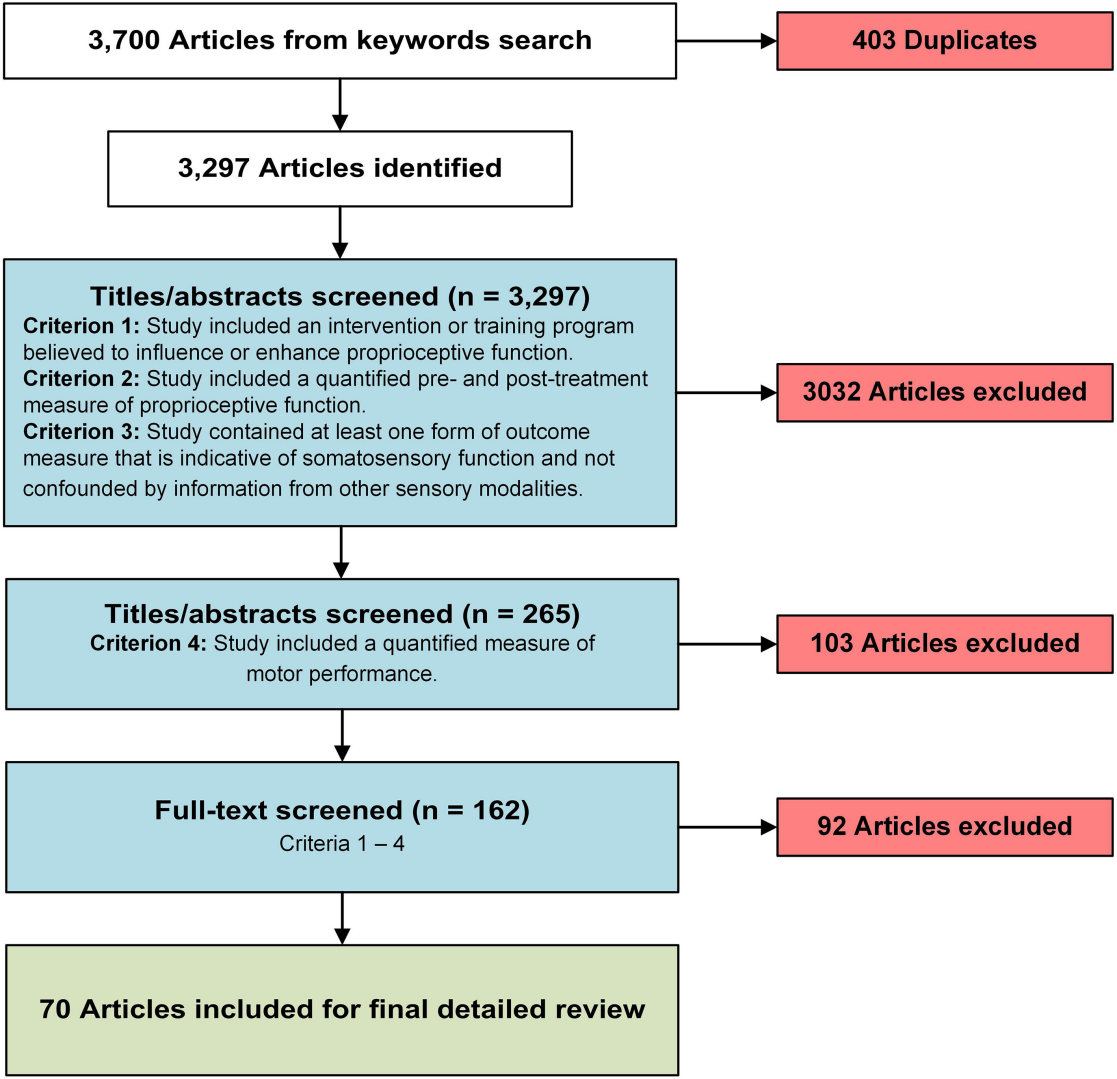
A flow chart of the article screening and selection process.

Search results were imported into Endnote (9) for deduplication and into Rayyan (10) for further screening. Authors LW, QH, and JS equally and independently reviewed titles, abstracts, and full texts for inclusion or exclusion. All titles and abstracts were reviewed by two authors. Pending disagreement between reviewers, the third author was employed to make the final decision of inclusion or exclusion. Full texts were reviewed by only one author. Articles in question were discussed by the full team and decisions were made with the full consensus of the team. Titles and abstracts were evaluated based on inclusion criteria #1 and #2 and full texts were evaluated based on all four inclusion criteria (see Figure 1).

Following Aman et al. (1), three quantitative evaluation measures were obtained for each study in the current review: First, within-group pre- to post-treatment scores were converted to a percentage of change for both proprioceptive and motor measures. These scores were most consistently reported data among all studies and allow comparison across a range of training protocols, outcome measures, and disease entities. The following formula was used to calculate percent change:


$$\begin{array}{l} \%\;change=\;\frac{posttest\;score-pretest\;score}{pretest\;score}\;\times \;100 \end{array}$$

Second, the *physiotherapy evidence database* (PEDro) scale was applied to measure the external and internal validity and the study interpretability (11). Only studies with a comparable control group were scored (see Table 1). Third, Cohen's *d* was calculated to quantify effect size (i.e., the standardized difference between two means). For those articles where sufficient data were provided, Cohen's *d* effect size was calculated for both between-group (e.g., control vs. intervention) (see Table 2) and within-group (pre-post treatment effect of a single group) comparisons (see Table 3). To calculate Cohen's *d* for between groups (d_s_) the following formula was used:


$$\begin{array}{l} d_{s}=\;\frac{{\bar{X}}_{1}-{\bar{X}}_{2}}{\sqrt{\frac{\left(n_{1}-1\right)SD_{1}^{2}+\left(n_{2}-1\right)SD_{2}^{2}}{n_{1}+n_{2}-2}}} \end{array}$$

**Table 1:** Summary of all reviewed studies, categorized by intervention type.

**Disease entity**	**References**	**Sample size**	**Intervention**	**Anatomical location**	**Proprioceptive measures**	**Motor measures**	**PEDro**
**Active movement/balance training**							
ACL injury	Büyükturan et al. (12)	*n*_1_ = 29 *n*_2_ = 29	Multi-joint movement	Whole body	Active JPSE	**International Knee Documentation Committee Questionnaire**, Isokinetic knee ext/flex strength, Lysholm Knee Score	6
	Ordahan et al. (13)	*n*_1_ = 20 *n*_2_ = 16	Multi-joint movement	Whole body	Passive JPSE	**Lysholm Knee Score**, Tegener Activity Score	4
	Peultier-Celli et al. (14)	*n*_1_ = 32 *n*_2_ = 35	Multi-joint movement	Knee	Active JPSE	6 Minute Walk Test, CoP displacement, International Knee Documentation Committee Questionnaire, Knee Injury and Osteoarthritis Outcomce Score, Lysholm Knee Score, muscle strength, Tegener Activity Score	7
	Zult et al. (15)	*n*_1_ = 22 *n*_2_ = 21	Multi-joint movement	Whole body	JPSE	**Knee ext muscle strength**, single leg balance time	5
	Wang (16)	*n* = 10	Single-joint movement	Lower extremity	Active JPSE	**Knee ext muscle strength**	3
Ankle sprain	Lazarou et al. (17)	*n*_1_ = 10 *n*_2_ = 10	Balance training	Whole body	Active JPSE	Ankle inversion/eversion and **dorsiflexion/plantarflexion strength**	7
Cerebral Palsy	El Shemy (18)	n_1_ = 15 *n*_2_ = 15	Multi-joint movement	Whole body	Active JPSE	**Modified TUG**, Pediatric Balance Scale	7
	El-Gohary et al. (19)	*n*_1_ = 24 *n*_2_ = 24	Balance training	Whole body	Passive JPSE	**Gross Motor Function Measure-88**, Pediatric Balance Scale	4
Chronic ankle instability	Lee et al. (20)	*n*_1_ = 15 *n*_2_ = 15	Multi-joint movement	Lower extremity	Active JPSE	Cumberland ankle instability tool, **SI**	7
	Lee et al. (21)	*n*_1_ = 21 *n*_2_ = 20	Multi-joint movement	Whole body	Active JPSE	**Functional Limitation Assessment Motor Functions**, Motor Limitation Assessment of Dynamic Balancing Ability, Sensory Limitation Assessment of Static Balancing Ability	2
	Hanci et al. (22)	*n* = 13	Single-joint movement	Lower leg	Active/Passive JPSE, Joint position sense detection threshold	**Ankle eversion/dorsiflexion strength**	N/A
Deaf	Zarei and Norasteh (23)	*n*_1_ = 10 *n*_2_ = 10	Balance training	Whole body	Active JPSE	**Single leg stance**	6
Diabetes II	Cavegn and Riskowski (24)	*n* = 8	Multi-joint movement	Whole body	Active JPSE	CoP displacement and sway area, **Senior Fitness Test**	N/A
Healthy adults	Alikhani et al. (25)	*n*_1_ = 12 *n*_2_ = 10	Multi-joint movement	Whole body	Active JPSE	**Lower Quadrant YBT**	6
	El-Gohary et al. (26)	*n*_1_ = 30 *n*_2_ = 30	Balance training	Lower extremity	Active JPSE	**SI**	6
	Hu et al. (27)	*n* = 12	Single-joint movement	Forearm	Active JPSE	**Writing time evaluation**	4
	Kalaycioglu et al. (28)	*n* = 24	Multi-joint movement	Whole body	Active JPSE	Hip flex/ext muscle strength**, Tracking Trajectory Test**, Sit-and-reach Test, Vertical Jump Test	N/A
	Lin and Karduna (29)	*n*_1_ = 18 *n*_2_ = 18	Single-joint movement	Shoulder	Active JPSE	**Shoulder girdle muscle strength**	5
	Lopes et al. (28)	*n*_1_ = 37 *n*_2_ = 34	Multi-joint movement	Whole body	Active JPSE	CoP displacement, YBT	4
	Minshull et al. (30)	*n*_1_ = 9 *n*_2_ = 9	Single-joint movement	Lower extremity	Active JPSE, Force reproduction error	Knee flex muscle strength, **passive ROM hip**	4
	Park et al. (31)	*n*_1_ = 21 *n*_2_ = 21	Multi-joint movement	Knee	JPSE	ROM and **stability knee**	5
	Peer and Gleeson (32)	*n*_1_ = 12 *n*_2_ = 11	Balance training	Whole body	JPSE, Force reproduction error	Knee flex muscle strength	4
	Pérez-Silvestre et al. (33)	*n*_1_ = 17 *n*_2_ = 17	Multi-joint movement	Whole body	Active JPSE	Counter-movement jump	8
	Sohn and Kim (34)	*n* = 18	Balance training and multi-joint movement	Lower extremity	Passive JPSE	Center of gravity velocity, CoP displacement and sway area, ground reaction forces, Knee and ankle flex/ext muscle strength, sliding heel velocity, slip distance	5
	Walsh (35)	*n* = 10	Single-joint movement	Lower extremity	Active JPSE	Knee flex/ext muscle strength	6
	Winter et al. (36)	*n*_1_ = 14 *n*_2_ = 14	Balance training and multi-joint movement	Lower leg	Joint position sense detection threshold	CoP displacement, SI	3
Healthy elderly adults	Chittrakul et al. (37)	*n*_1_ = 36 *n*_2_ = 36	Balance training and multi-joint movement	Whole body	JPSE	**CoP displacement**, knee ext muscle strength	8
	Lee et al. (38)	*n*_1_ = 313 *n*_2_ = 303	Multi-joint movement	Whole body	Active JPSE	**CoP displacement**, knee flex/ext muscle strength, reaction time, TUG	6
	Merom et al. (39)	*n*_1_ = 279 *n*_2_ = 251	Multi-joint movement	Whole body	Active JPSE	Physical Performance Assessment, Trail Making Tests	4
	Zheng et al. (40)	*n*_1_ = 50 *n*_2_ = 50	Balance training	Whole body	Passive JPSE	BBS, CoP displacement and sway area	8
	Wooten et al. (41)	*n*_1_ = 15 *n*_2_ = 15	Multi-joint movement	Whole body	Passive JPSE, Joint position sense detection threshold	Balance Error Scoring System, **dynamic motion analysis score**, Tinetti gait and balance assessment, leg press peak power	3
Hypermobility syndrome	Liaghat et al. (42)	*n* = 12	Single-joint movement	Shoulder	Active JPSE	ROM, Shoulder joint mobility and laxity tests, **scaption** and IR/ER **muscle strength**	N/A
	Daman et al. (43)	*n*_1_ = 12 *n*_2_ = 12	Multi-joint movement	Whole body	Active JPSE	**36-Item Short Form Health Survey – Physical functioning**	5
Infraspinatus muscle atrophy	Salles et al. (44)	*n*_1_ = 18 *n*_2_ = 18	Single-joint movement	Shoulder	Active JPSE, Joint position sense detection threshold	**Shoulder ER muscle strength**	2
Knee Osteoarthritis	Gezginaslan et al. (45)	*n* = 39	Multi-joint movement	Lower extremity	Active JPSE	6 Meter Walk Test, BBS, Five-minute sit-to-stand Test, **knee flex/ext muscle strength**, ROM, TUG, WOMAC	N/A
	Knoop et al. (46)	*n* = 159	Multi-joint movement	Whole body	Joint position sense detection threshold	Knee flex/ext muscle strength, **WOMAC**	8
	Kumar, Kumar, and Kumar (47)	*n* = 44	Multi-joint movement	Lower extremity	Active JPSE	**WOMAC**	7
Multiple Sclerosis	Moghadasi et al. (48)	*n*_1_ = 19 *n*_2_ = 15	Multi-joint movement	Whole body	Active JPSE	TUG, 10 Meter Walk Test, 2 Minute Walk Test, **5-time sit-to-stand**, knee flex/ext muscle strength	4
Neck pain	Shiravi et al. (49)	*n* = 135	Multi-joint movement	Upper body	Active JPSE	Scapular upward rotation muscle strength	7
Parkinson's Disease	Daneshvar et al. (50)	*n*_1_ = 10 *n*_2_ = 10	Multi-joint movement	Whole body	JPSE	Muscle strength, peak muscle torque, **ROM knee**	7
	Elangovan et al. (4)	*n*_1_ = 13 *n*_2_ = 13	Single-joint movement	Wrist	Joint position sense detection threshold	**Cumulative spatial error**, handwriting task, line tracing task	3
	Peterka et al. (51)	*n*_1_ = 30 *n*_2_ = 15	Multi-joint movement	Whole body	JPSE	Handwriting speed, Movement Disorder Society-Sponsored Revision of the Unified Parkinson's Disease Rating Scale-III, Nine-hole peg test, Spiral drawing	2
Stroke	Herrnstadt et al. (52)	*n* = 8	Multi-joint movement	Upper extremity	Active JPSE	Fugl-Meyer Assessment, **Wolf Motor Function Test**	N/A
	Ingemanson et al. (53)	*n* = 30	Single-joint movement	Finger	Passive JPSE	Action Arm Research Test, **Box and Blocks Test**, Finger Tapping Test, Fugl-Meyer Assessment, Nine-hole peg Test	N/A
Subacromial impingement syndrome, tennis elbow	Babaei-Mobarakeh et al. (54)	*n*_1_ = 15 *n*_2_ = 15	Multi-joint movement	Shoulder and wrist	Active JPSE	Shoulder, grip and wrist strength; **Upper Quarter YBT**	7
	Dilek et al. (55)	*n*_1_ = 31 *n*_2_ = 30	Single-joint movement	Shoulder	Joint position sense detection threshold, Active/ Passive JPSE	**American Shoulder and Elbow Surgeons Shoulder Score**, shoulder ABD/ER muscle strength, ROM	8
	Boarati et al. (56)	*n*_1_ = 50 *n*_2_ = 50	Single-joint movement	Shoulder	Active JPSE	Shoulder ABD strength	4
Total knee or hip replacement	Eymir et al. (57)	*n*_1_ = 58 *n*_2_ = 55	Single-joint movement	Knee	Active JPSE	10 Meter Walk Test, Iowa Ambulation Velocity Scale, TUG	4
	Moutzouri et al. (58)	*n*_1_ = 26 *n*_2_ = 25	Multi-joint movement	Lower extremity	Active JPSE	**Knee Outcome Survey Activities of Daily Living Scale**, TUG, single leg stance	7
	Pohl et al. (59)	*n* = 58	Balance training	Whole body	Active JPSE	CoP displacement, Lequesne Algofunctional Index, step length, walking velocity	4
**Passive movement training**							
Spinal cord injury	Qaiser et al. (60)	*n*_1_ = 15 *n*_2_ = 10	Passive movement	Lower extremity	Passive JPSE	**Precision stepping task**	2
**Somatosensory stimulation training**							
ACL injury	Fu et al. (61)	*n*_1_ = 24 *n*_2_ = 24	Whole body vibration	Whole body	Passive JPSE	**Knee flex/ext muscle strength**, Shuttle Run and Carioca Tests, Single Legged and Triple Hop Tests, ROM, SI	5
	Liu et al. (62)	*n* = 48	Kinesio taping	Knee	Active JPSE	**Tibial anteposterior shift**, Lysholm Knee Score, modified Star Excursion Balance Test, Single-hop distance	N/A
Cerebral Palsy	Ko et al. (63)	*n*_1_ = 12 *n*_2_ = 12	Whole body vibration	Whole body	Active JPSE	Gait speed, SI, step length, **step width**	4
Healthy adults	Hiroshige et al. (64)	*n*_1_ = 9 *n*_2_ = 18	Whole body vibration	Whole body	Active JPSE	CoP displacement, **single leg stance time with eyes closed**	2
Healthy adults	Lee et al. (65)	*n*_1_ = 10 *n*_2_ = 10	Foam rolling	Lower extremity	Active JPSE	**Knee ext/flex muscle strength**, ROM knee, YBT	N/A
Healthy adults	Mustafa et al. (66)	*n* = 30	Massage	Hand	Force reproduction error	Grip strength	4
Healthy adults	Naderi et al. (67)	*n*_1_ = 40 *n*_2_ = 40	Foam rolling	Lower extremity	Active JPSE, Joint position sense detection threshold	Knee ext muscle strength	4
Healthy adults	Weerakkody and Allen (68)	*n* = 14	Kinesio taping	Shoulder	Active JPSE	**Shoulder flex/ext muscle strength**	N/A
Knee Osteoarthritis	Cho et al. (69)	*n*_1_ = 23 *n*_2_ = 23	Kinesio taping	Knee	Active JPSE	Pain-free active ROM	8
Stroke	Kattenstroth et al. (70)	*n*_1_ = 23 *n*_2_ = 23	Electrical stimulation	Hand	Active JPSE	Motor performance index (grip strength and Nine-hole peg test)	5
**Somatosensory discrimination training**							
Cerebral Palsy	McLean et al. (71)	*n*_1_ = 7 *n*_2_ = 10	Somatosensory discrimination	Hand	Active JPSE	Assisting Hand Assessment, Box and Block Test, Goal Attainment Scaling	4
**Combined/multiple system training**							
ACL relaxation	Wei et al. (72)	*n*_1_ = 16 *n*_2_ = 15	Somatosensory stimulation and active movement training	Lower extremity	Passive JPSE	Lysholm Knee Score, Tegener Activity Score	6
Ankle sprain	Alahmari et al. (73)	*n*_1_ = 20 *n*_2_ = 20	Somatosensory stimulation and active movement training	Lower leg	Active JPSE	Active ROM ankle, Foot and Ankle Disability Index, knee-to-wall distance, muscle strength, Star Excursion Balance Test	7
Multiple Sclerosis	Lee et al. (74)	*n* = 7	Passive stretching and active exercise	Lower leg	Joint position sense detection threshold	10 Meter Walk test, 6 Minute Walk Test, active and passive ankle ROM, BBS, Fugl-Meyer Assessment, ankle dorsiflexion/plantarflexion strength, Modified Ashworth Scale, Selective Control Assessment of the Lower extremity, TUG	N/A
Plantar Fasciitis	Akinoglu et al. (75)	*n*_1_ = 18 *n*_2_ = 18	Somatosensory stimulation and active movement training	Lower leg	Passive JPSE	American Orthopaedic Foot and Ankle Society Ankle-Hindfoot Score, Functional Foot Index, Functional Reach Test, single leg stance	7
Stroke	Lim (76)	*n*_1_ = 19 *n*_2_ = 18	Somatosensory stimulation and active movement training	Whole body	Active JPSE	SI	4
Total knee replacement	Wozniak-Czekierda et al. (77)	*n*_1_ = 51 *n*_2_ = 60	Somatosensory stimulation and active movement training	Whole body and knee	Active JPSE	6 Minute Walk Test, CoP displacement and sway area	4
**Meditation**							
Healthy elderly adults	Chatutain et al. (78)	*n*_1_ = 29 *n*_2_ = 29	Walking meditation	Whole body	Active JPSE	BBS, Functional Reach Test, TUG	6
Parkinson's Disease	Cherup et al. (79)	*n*_1_ = 15 *n*_2_ = 18	Yoga meditation	Whole body	Passive JPSE, Joint position sense detection threshold	BBS, CoP displacement, time on platform, Tinetti gait and balance assessment, TUG	5

*n_1_, sample size experimental group; n_2_, sample size control group; n, sample size; ABD, abduction; BBS, Berg Balance Scale; CoP, Center of Pressure; ER, external rotation ext, extensor; flex, flexor; ROM, Range of Motion; SI, Stability Index; TUG, Timed Up and Go; WOMAC, The Western Ontario and McMaster Universities Arthritis Index; YBT, Y-Balance Test; N/A, not applicable. Outcome measures with the largest pre-post-treatment effect are displayed in bold. PEDro scores are calculated for studies that included a control group*.

**Table 2:** Effect sizes for between-group comparisons.

**References**	**Disease entity**	**Outcome measure**	**Sample size**		**SD_**pooled**_**	**Cohen's d**	**95% CI**	
			**Control**	**Training**			**LL**	**UL**
**Active movement/balance training**								
Alikhani et al. (25)	Healthy adults	Active JPSE	10	12	1.497	1.576	0.717	2.795
		Lower Quadrant Y-Balance test			7.596	1.438	0.586	2.618
Büyükturan et al. (12)	ACL injury	Active JPSE	29	29	1.265	1.344	0.811	1.982
		International Knee Documentation Committee-2000			16.415	0.256^†^	0.794^†^	−0.262^†^
Chittrakul et al. (37)	Healthy elderly adults	JPSE	36	36	1.3	2.039	1.517	2.684
		CoP displacement			364.964	1.552	1.061	2.139
Daman et al. (43)	Hypermobility syndrome	Active JPSE	12	12	2.508	1.176	0.373	2.225
		SF-36 - Physical functioning			9.47	1.197^†^	0.393^†^	2.251^†^
Daneshvar et al. (50)	Parkinson's Disease	JPSE	10	10	2.874	0.967	0.092	2.094
		Range of motion knee			12.855	1.262^†^	0.382^†^	2.467^†^
Eymir et al. (57)	Total knee replacement	Active JPSE (day of discharge)	55	58	2.709	0.997	0.62	1.412
		TUG (day of discharge)			26.74	0.43	0.061	0.816
El Shemy (18)	Cerebral Palsy	Active JPSE	15	15	3.057	1.963	1.191	3.043
		modified TUG			2.876	1.878	1.114	2.938
El-Gohary et al. (26)	Healthy adults	Active JPSE	30	30	1.487	0.874	0.366	1.45
		Stability Index (AP direction)			1.523	1.773	1.224	2.453
El-Gohary et al. (19)	Cerebral Palsy	Passive JPSE	24	24	1.371	0.766	0.2	1.406
		Gross motor function (walking)			4.393	0.824^†^	0.257^†^	1.471^†^
Hu et al. (27)	Healthy adults	Active JPSE	12	12	1.051	3.33	2.98	5.02
		Writing time			6.912	1.071	0.27	2.095
Kumar et al. (47)	Knee osteoarthritis	Active JPSE	22	22	1.961	1.117	0.518	1.833
		Western Ontario and McMaster Universities Osteoarthritis Index			2.971	1.027	0.431	1.731
Lee et al. (20)	Chronic ankle instability	Active JPSE	15	15	N/A	eta^2^ = 0.14^*^	N/A	N/A
		Stability Index			N/A	eta^2^ = 0.192^*^	N/A	N/A
Merom et al. (39)	Healthy elderly adults	Active JPSE	147	275	N/A	not sig.	N/A	N/A
		Center of pressure displacement			165.154	−0.279^†^	−0.482^†^	0.078^†^
Salles et al. (44)	Infraspinatus muscle atrophy	Active JPSE	18	18	0.544	2.595	1.822	3.697
		Shoulder external rotation peak torque			6.017	1.612^†^	0.929^†^	2.505^†^
Shiravi et al. (49)	Neck pain	Active JPSE	44	43	0.523	5.736	4.894	6.85
		Isometric scapular upward rotation muscle strength			N/A	not sig.	N/A	N/A
Sohn and Kim (34)	Healthy adults	Passive JPSE	6	6	N/A	not sig.	N/A	N/A
		Isokinetic knee extension muscle strength^†^			7.663	2.505	1.272	4.945
Zarei and Norasteh (23)	Deaf	Active JPSE	10	10	N/A	N/A	N/A	N/A
		Single leg stance (eyes closed)			2.155	1.949^†^	1.024^†^	3.367^†^
**Somatosensory stimulation training**								
Cho et al. (69)	Knee osteoarthritis	Active JPSE	23	23	4.008	2.67	1.969	3.629
		Pain-free active range of motion			10.847	1.872^†^	1.244^†^	2.684^†^
Fu et al. (61)	ACL injury	Passive JPSE	20	19	N/A	not sig.	N/A	N/A
		Stability Index (AP direction)			1.843	0.873	0.245	1.608
Naderi et al. (61)	Healthy adults	JPSE	40	40	N/A	0.93^*^	N/A	N/A
		Isokinetic knee extension muscle strength			N/A	0.66^*^	N/A	N/A
**Somatosensory discrimination training**								
McLean et al. (71)	Cerebral Palsy	Active JPSE	10	8	N/A	N/A	N/A	N/A
		Goal Attainment Scaling			N/A	0.79^*^	N/A	N/A
**Combined/multiple system training**								
Akinoglu et al. (75)	Plantar Fasciitis	Passive JPSE	18	18	1.916	0.819	0.166	1.581
		American Orthopedic Foot and Ankle Society Ankle-Hind foot Score			11.637	1.308^†^	0.642^†^	2.145^†^
Alahmari et al. (73)	Ankle Sprain	Passive JPSE	20	20	0.985	2.234	1.533	3.189
		Knee-to-wall distance (cm)			1.02	2.942^†^	2.164^†^	4.049^†^
Lim (76)	Stroke	Active JPSE	15	15	2.743	0.558^†^	0.016^†^	1.367^†^
		Stability Index			0.78	0.577^†^	0.141^†^	1.389^†^
Wei et al. (72)	ACL injury	Passive JPSE	15	16	N/A	N/A	N/A	N/A
		Lysholm Knee Score			7.36	0.666^†^	0.039^†^	1.475^†^
**Meditation**								
Chatutain et al. (78)	Healthy elderly adults	Active JPSE	29	29	1.681	1.071	0.549	1.677
		Functional Reach Test			5.064	2.113^†^	1.531^†^	2.857^†^
Cherup et al. (79)	Parkinson's Disease	Joint position sense detection threshold	18	15	N/A	0.72^*^^†^	0.1^*^^†^	1.4^*^^†^
		Tinetti Score			3.141	0.77^*^	0.1^*^	1.5^*^

*For studies that applied multiple proprioceptive or motor measures, respectively, only the proprioceptive and motor measure with the largest improvement between pre- and post-intervention measures is shown*.

† *Direction of effect size was converted that increment indicates improvement*.

* *Effect size reported in study (*Cohen's d* unless otherwise specified)*.

*AP, anterior-posterior; JPSE, Joint position sense error; N/A, data not available; TUG, Timed Up and Go Test*.

**Table 3:** Effect sizes for within-group comparisons.

**References**	**Disease entity**	**Sample size**	**Outcome measures**	**Cohen's d**
**Active movement/balance training**				
Cavegn and Riskowski (24)	Diabetes II	8	Active JPSE	1.837
			Chair sit-and-reach (Senior Fitness Test)	1.187
El-Gohary et al. (19)	Cerebral Palsy	24	Passive JPSE	2.339^*^
			Paedeatric Balance Scale	0.872^*^^†^
Elangovan et al. (4)	Parkinson's Disease	13	Discrimination threshold	1.24
			Spatial error	1.1
Kalaycioglu et al. (28)	Healthy adults	24	Active JPSE	0.659
			Lower Quadrant Y-Balance test	0.986
Lopes et al. (80)	Healthy adults	37	Active JPSE	not sig.
			Lower Quadrant Y-Balance test	eta^2^ = 0.130
Moghadasi et al. (48)	Multiple Sclerosis	16	Active JPSE	eta^2^ = 0.223
			Five-times-sit-to-stand	eta^2^ = 0.440
Moutzouri et al. (58)	Total knee replacement	26	Active JPSE	1.8
			Knee Outcome Score Activities of Daily Living	5.5
Park et al. (31)	Healthy adults	21	JPSE	1.438^*^
			Stability index	0.615^*^
Peer and Gleeson (32)	Healthy adults	12	Force reproduction error	0.45
			Isometric knee flexion muscle strength	not sig.
Salles et al. (44)	Infraspinatus muscle atrophy	18	Active JPSE	2.942^†^
			Isokinetic shoulder external rotation peak torque	2.782
Wooten et al. (41)	Healthy elderly adults	6	Motion detection, passive JPSE	not sig.
			Dynamic balance performance	1.238
Zarei and Norasteh (23)	Deaf	10	Active JPSE	2.83
			Single leg stance	1.89
**Somatosensory stimulation**				
Kattenstroth et al. (70)	Stroke	23	JPSE (weight of errors)	0.37
			N/A	N/A
Lee et al. (65)	Healthy adults	30	Active JPSE	0.4
			Quadriceps peak torque	0.47
**Somatosensory discrimination training**				
McLean et al. (14)	Hemiplegic Cerebral Palsy	8	Active JPSE	0.54
			Goal Attainment Scaling	N/A

*For studies that applied multiple proprioceptive or motor measures, respectively, only the proprioceptive and motor measure with the largest improvement between pre- and post-intervention measures is shown*.

† *Direction of effect size was converted that increment indicates improvement*.

* *Effect size calculated with t-value, otherwise effect size reported in study (*Cohen's d* unless otherwise specified)*.

*JPSE, Joint position sense error; N/A, data not available*.

where *d*_*s*_ refers to the standardized mean difference between two independent groups of observations, ${\bar{X}}_{1}$ and ${\bar{X}}_{2}$ are the means of group one and two, respectively, *n*_1_ and *n*_2_ are the sample sizes of group one and two, respectively, and *SD*_1_ and *SD*_2_ are the standard deviations of group one and two, respectively. The numerator is mean difference of the two groups of observations and the denominator is the pooled standard deviation (81). To calculate the within-group Cohen's *d* (d_z_), the following formula was used following Rosenthal (82):


$$\begin{array}{l} d_{z}=\;\frac{t}{\sqrt{n}} \end{array}$$

where d_z_ refers to the within-subject standardized mean difference, *t* is the *t*-value of the group measures and *n* is the sample size. If a study had multiple proprioceptive or motor measures, respectively, effect sizes were only calculated for the largest difference between post-intervention measures between groups, or the largest percentage improvement between pre-and post-intervention measures for within-group effect sizes, respectively.

## Results

### Initial Search Results and Final Included Studies

The initial search yielded 3,700 articles. Of those, 403 articles were duplicates, resulting in a final total of 3,297 identified studies. Subsequently, three authors reviewed all titles and abstracts independently and applied three specific inclusion criteria (see Figure 1). A total of 264 articles met those criteria. In a next step, a fourth criterion was applied requiring that the reported research included at least one motor outcome measure that isolated sensory from motor improvements due to the intervention. This resulted in 161 articles that met all four criteria based on the review of title and abstract. Finally, all four selection criteria were applied again on the full-text articles, yielding 70 articles that were included in this systematic review. The main reason for exclusion of articles in this final step was wording in the title and abstract that indicated that all inclusion criteria were met, while review of the full text showed that this was not the case. For example, in the abstract, many studies indicated the use of a proprioceptive outcome measure but did not specify it. The full text subsequently revealed that the measure did not meet our criteria, which led to the exclusion in the final step. The cumulative sum of participants in the included studies is 4,068, with study sample sizes ranging from 7 to 616 participants.

### Classification by Outcome Measures

We first categorized the reported proprioceptive and motor outcome measures. In general, measures indicative of proprioceptive function can be broadly classified into *somatosensory* measures that were based on passive movements of the limb or body, and *somatosensory-motor* measures during active movement. This distinction is important as *somatosensory-motor* measures reflect the contribution of somatosensory as well as voluntary motor control processes, while *somatosensory* measures reflect solely the processing of somatosensory signals.

### Classification by Proprioceptive Outcome Measures

**Figure 2:**
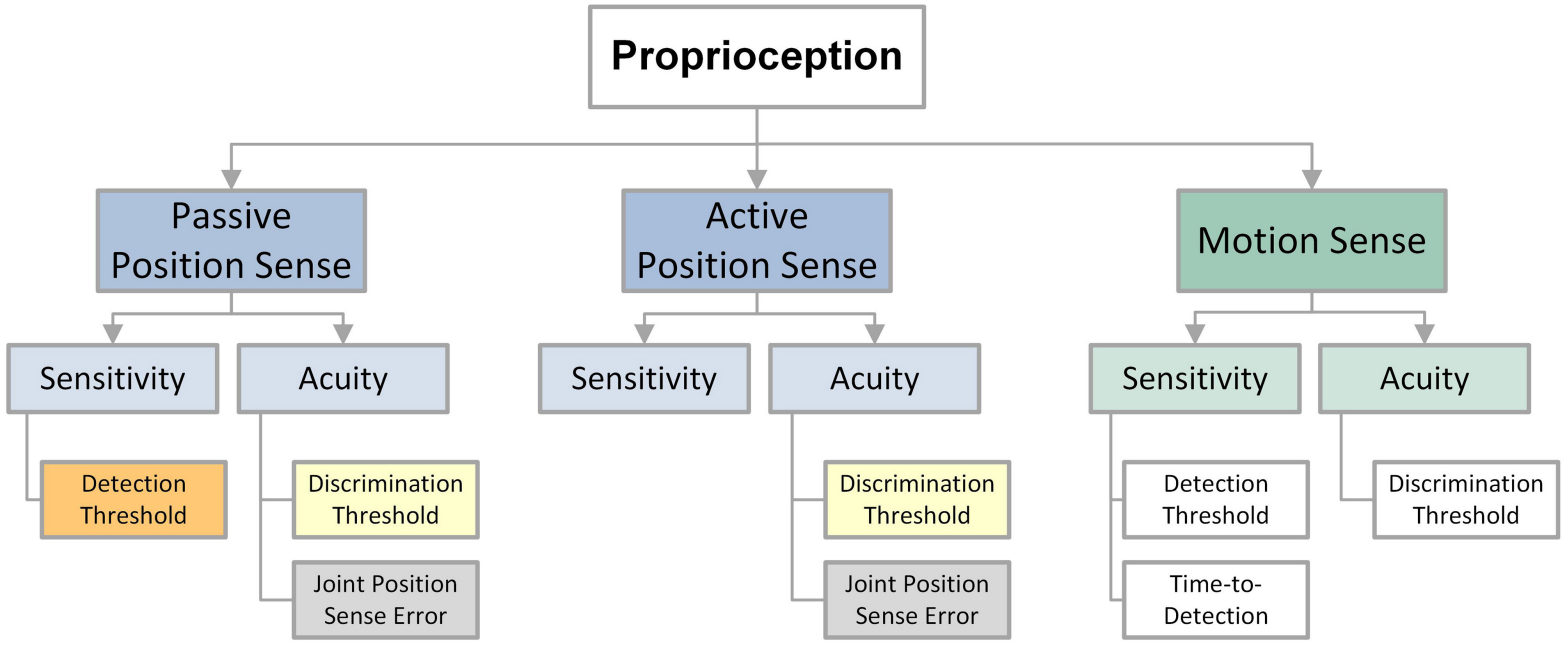
Overview over position and motion related proprioceptive outcome measures.

We separated proprioceptive outcome measures into four categories according to what aspect of proprioceptive function it sought to measure: *force reproduction error, joint position sense error (JPSE), joint position sense detection threshold*, and *joint position sense discrimination threshold*. Studies that required participants to match a target force typically reported a force reproduction error as a measure of proprioceptive function. Testing paradigms that required the matching of a previously experienced joint position or the concurrent matching across two homologous joints often derived a *joint position sense error* (JPSE). Such joint matching was either performed actively by the participant, or the joint(s) were passively moved by an apparatus. Few studies applied psychophysical methods to obtain *joint position sense detection* or *joint position sense discrimination* thresholds. These methods require participants to make verbal judgments on joint position(s), which are then used to fit a stimulus-response function. A joint position sense detection threshold is an estimate of the minimum perceivable change in position and serves as a measure of proprioceptive sensitivity. A joint position sense discrimination threshold, or the *just noticeable difference* (JND) between two perceived joint positions represents a measure of proprioceptive acuity. Active movement-based JPSE and force reproduction error are considered somatosensory-motor measures. Passive movement-based JPSE, joint position sense detection and discrimination thresholds represent somatosensory measures (Figure 2).

**Figure 3:**
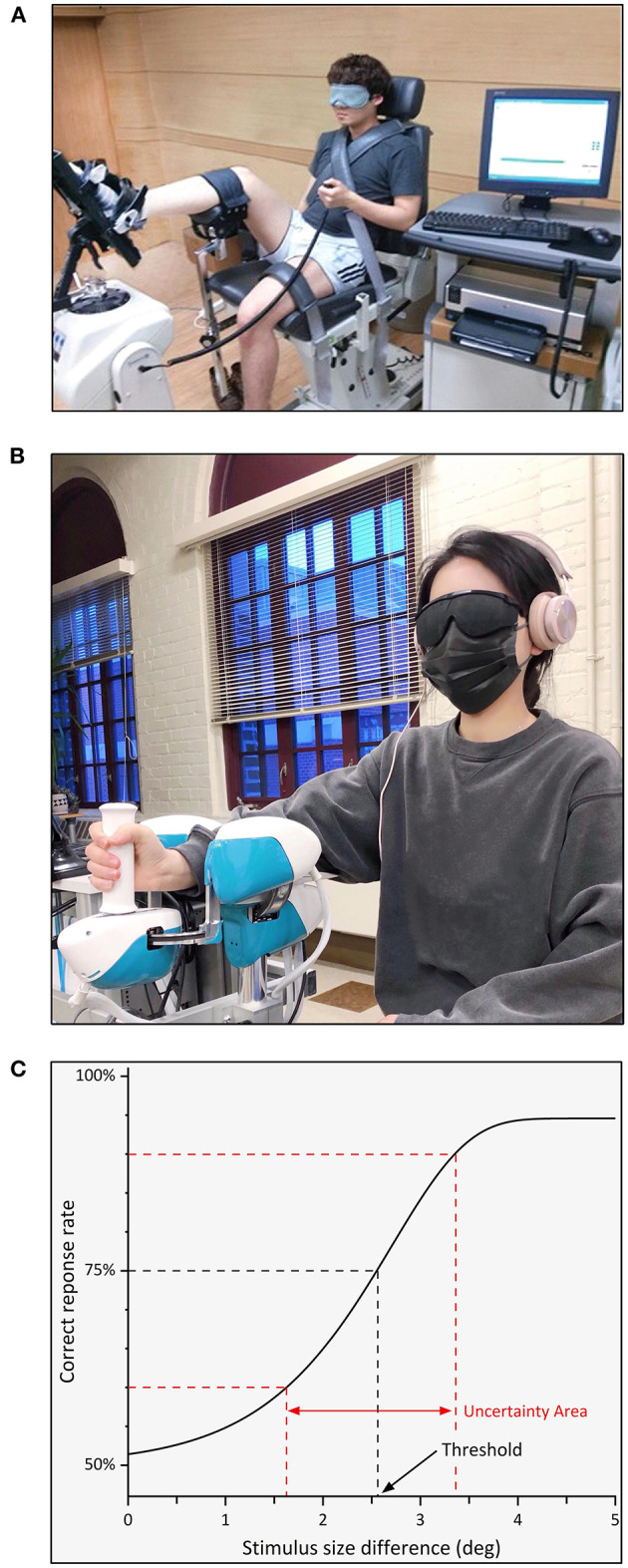
Two examples of an experimental setup to obtain proprioceptive outcome measures and position sense discrimination threshold estimation based on participant responses. Visual and acoustic stimuli are typically blocked during these assessments. **(A)** An ipsilateral ankle joint matching task uses modified isokinetic device. The ankle is passively rotated by the device to a target position. In a second displacement participants stop the passive motion when they perceive to have reached the previous position (20). **(B)** A robotic device that passively displaces the wrist joint during a joint position discrimination task. The robot rotates the wrist in two consecutive trials from a neutral to a standard or a comparison position. The participant verbally responds to which position is further away from the neutral position. **(C)** Using verbal responses and experienced proprioceptive stimuli as inputs, a proprioceptive acuity function is fitted. The corresponding joint position sense discrimination threshold reflects the 75% correct response rate. The uncertainty area between the 60-90 percentiles is a measure of precision, reflecting a person's reliability in making consistent perceptual judgments.

An example of an ipsilateral, active joint position matching task (20) is illustrated in Figure 3A. Using a Biodex system, a participant's foot was passively moved to a target position (e.g., 15° eversion) and then returned to the starting position. Subsequently, the participant actively matched the target. After three trials, the active JPSE was calculated as the average difference between the target and matched positions. An example of a psychophysical assessment of proprioceptive function is depicted in see Figure 3B. This study used a robotic device to obtain a wrist *joint position sense discrimination threshold* (4). The blindfolded participant's wrist was moved by the robot successively to two distinct wrist joint positions (a fixed *standard* and a variable *comparison* position) and then verbally indicated, which position was farther from the starting position (first or second). Based on the participant's response, the comparison position was adjusted (e.g., increased or decreased). After 30 trials, a wrist position sense discrimination threshold was estimated as the angular difference at which the participant achieved 75% correct response rate (see Figure 3C).

Several studies attempted to determine joint motion sense sensitivity (36, 41, 44, 46, 55, 74, 79), reporting the angular displacement or time duration to perceive a passive movement at a single slow velocity (velocity range: 0.3–1.5 deg/s). Because these studies only applied a single low velocity, their approach is identical to those studies that determined joint position sense. Thus, for the purpose of this review, we categorized them as belonging to the group of position sense studies. JPSE was the most commonly used proprioceptive outcome measure (65 out of 70 studies); most of those studies (48 out of 65) used active JPSE. Compared to measures such as force reproduction error or joint position sense detection threshold, JPSE measures do not require additional equipment or automated movement of a limb. It is therefore unsurprising that measurement of JPSE has become widely used in clinical and research settings in order to quantify proprioceptive function (83, 84).

### Classification by Motor Outcome Measures

The reported motor outcome measures can be grouped into three broad categories: (1) *clinical rating scales*, (2) *joint-specific measures*, or (3) *whole-body postural stability measures*. Clinical rating scales included clinical measures such as the reaching distance obtained in the Functional Reach Test to quantify balance. Joint-specific measures comprised joint kinematic variables such as movement time or range of motion (ROM), or kinetic variables such as peak force or torque. Whole-body postural control measures were typically based on center of pressure (CoP) data (e.g., sway area, sway path). Twenty studies utilized a variety of clinical scales as motor outcome measures. Another 25 studies reported joint-specific measures, and 25 different studies reported whole-body stability measures of motor performance (for details on used scales and specific assessments see Table 1). In addition, several studies obtained latencies and amplitudes of EMG signals (16, 30, 49).

### Effectiveness of Proprioceptive Training by Type of Intervention

**Figure 4:**
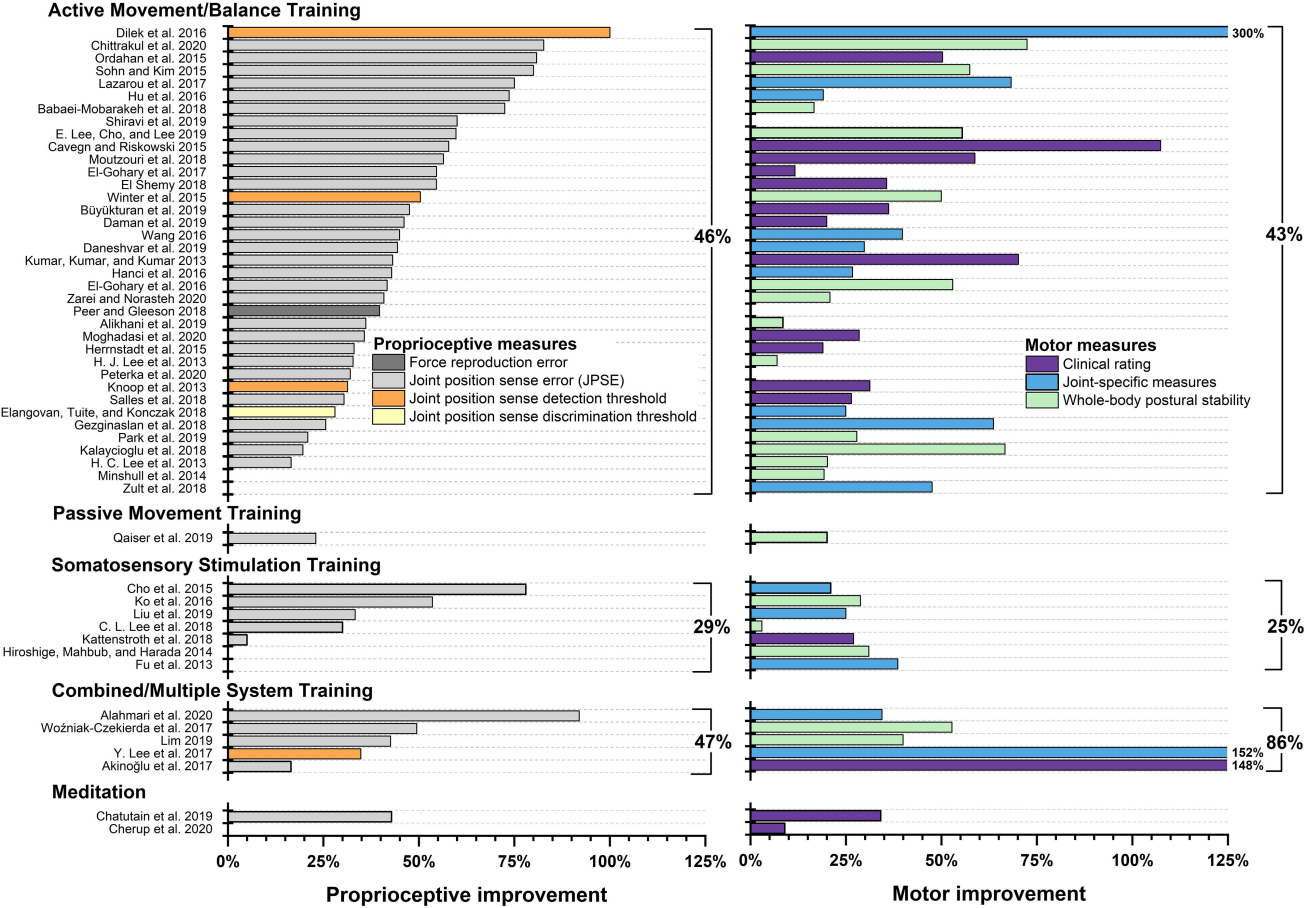
Treatment effects by intervention type. Studies not reporting exact data are not pictured and were not included in calculating the mean. For studies that applied multiple proprioceptive or motor measures, respectively, only the proprioceptive and motor measure with the largest pre-post intervention improvement is shown.

We grouped each included study into one of five categories according to the applied intervention: *Active Movement/Balance Training, Passive Movement Training, Somatosensory Stimulation Training, Somatosensory Discrimination Training, Combined/Multiple System Training*, and *Meditation* (85). Table 1 lists all studies by intervention category and summarizes relevant information on disease entity, type of intervention, trained limb or body system, proprioceptive and motor outcome measures, and PEDro score. Figure 4 displays treatment effects of proprioceptive and motor measures.

To gain an understanding of the potential training effectiveness, we calculated the effect sizes for between-group comparisons for studies that provided sufficient information (reported as Cohen's *d* unless otherwise specified; see Table 2. For Hedge's *g*, see Appendix B). Between-group effect sizes were calculated for those studies that showed no group differences at baseline. Table 3 shows effect sizes for within-group comparisons. Effect sizes (Cohen's *d*) for between-group comparisons ranged from 0.28 to 5.74 (see Table 2) and from 0.37 to 2.94 (see Table 3) for within-group comparisons. Four between-group and two within-group comparisons yielded small effect sizes (*d*_*z*_ ≥ 0.2, η^2^ ≥ 0.01) for proprioceptive and motor performance measures. Medium effect sizes (*d*_*z*_ ≥ 0.5, η^2^ ≥ 0.06) were seen in five and seven studies, respectively. The majority of studies revealed large effect sizes (*d*_*z*_ ≥ 0.8, η^2^ ≥ 0.14; 26 between-group and 30 within-group comparisons), indicating that the majority of interventions could induce changes in the reported outcome measures. There was no clear relationship between effect size and intervention type or outcome measure.

### Active Movement/Balance Training

The vast majority of reports (50/70 studies) investigated the effects of active movement and/or balance training. The following interventions were used: balance training (7 studies), active multi-joint movement (27 studies), active single-joint movement (13 studies), and balance training in combination with active multi-joint movement (3 studies). Balance interventions included single and double leg standing balance exercises on wobble boards, balance pads and cushions (17, 23, 26, 32, 34, 36, 37, 40, 59), seated balance exercises (23, 37), walking or jogging balance exercises (23, 40), and balance exercises using the Biodex balance system (19). Studies assessed predominantly healthy individuals, including younger (23, 26, 32, 36) and older adults (34, 37, 40). Clinical investigations focused on orthopedic populations such as individuals with ankle sprains (17) and after total knee or hip replacement (59), as well as children with cerebral palsy (19). The mean relative treatment effects for balance training were 58% (range 40–83%) for proprioceptive performance, and 48% (range 12–72%) for motor performance. It is noteworthy that balance training led to improvements in at least one proprioceptive or motor measure in all ten studies that assessed the impact of balance training on proprioception and motor performance. Eight studies showed improvements in both proprioceptive and motor performance. With respect to training duration, the two studies that led to improvement in only one measure used balance training for only 3 weeks (32, 59), compared to 6–12 weeks in the other eight studies.

Active multi-joint movements included whole-body training such as Tai Chi (12, 24), Yoga (41), whole-body strength and stabilization exercise, as well as lower limb rehabilitation (13, 15, 47), lower extremity strength and flexibility exercise, and upper limb strength, stability and flexibility exercise (49, 51, 52, 54) (see Table 1). Thirty studies fell into this subcategory. Training duration varied greatly between 3 weeks and 12 months but there was no apparent relationship between the total time spent training and the amount of improvement between pre-and post-intervention measures. The magnitude of the mean relative treatment effects was 48% (range 17–72%) for proprioceptive and 47% (range 7–107%) for motor performance outcome measures. Active multi-joint movements were used in orthopedic, neurological and non-clinical populations. The three studies that used a combination of balance and active multi-joint movement (34, 36, 37) showed large improvements in both proprioceptive (range 50–83%) and motor measures (range 50–72%) compared to studies that applied interventions of balance training or active multi-joint movements alone. These studies applied a combination of balance training and whole-body strength training (37) or lower limb strength training (34, 36) in healthy adults and adolescents.

In 13 studies, proprioceptive training focused on active single-joint movement. Eight of these studies reported improvements in at least one proprioceptive or motor measure, and six interventions led to improvements in both proprioceptive and motor performance. Effective single-joint interventions resulting in proprioceptive or motor function gains included active stretching of the hip (30), strength exercises for the shoulder (29, 44), active movement and proprioceptive exercises for the shoulder (55), strength exercises for the wrist (27), knee (16) and ankle (22), as well as robotic exercises for the wrist (4). Overall, mean relative treatment effects across all studies for proprioceptive performance were 53% (range 28–100%) and 148% (range 19–725%) for motor performance. Treatment duration tended to be shorter than for balance training and active multi-joint movement interventions, ranging from a single, 36 second bout of activity to 8 weeks, with no apparent relationship between intervention length and treatment effect. There is no clear evidence that specific joints are more sensitive to proprioceptive training interventions than others. It is noteworthy that of the five studies assessing isolated training of the shoulder (29, 42, 44, 55, 56), all but one report motor improvements (56), but only two report proprioceptive improvements at the end of the intervention (44, 55).

Overall, 43 of the 50 studies that used active movement and/or balance training showed improvement in at least one proprioceptive or motor measure post-treatment. In three studies, there was proprioceptive improvement but no change in motor performance (32, 49, 51); in eight studies, participants improved motor performance but not proprioception (15, 29, 30, 39, 41, 42, 48, 59), and in the remaining 32 studies, participants improved at least one proprioceptive and one motor measure (Figure 4). Treatment effects ranged from 17% (38) to 100% (55) for proprioceptive performance and from 6% (21) to 300% (55) for motor measures.

### Passive Movement Training

Only one study used passive movement training as the primary intervention. Qaiser et al. (60) investigated the effects of passive leg movements on proprioception and a spatial precision stepping task in 15 individuals with spinal cord injury and ten healthy controls. There was a 23% reduction in passive JPSE post-training (5.22° to 4.03°) across participants and a 20% reduction of precision error in the stepping task in the eight participants with spinal cord injury who were able to perform the task. In two studies, passive movement training was used as the control condition (30, 57). Eymir et al. (57) compared the effects of active heel slide exercises to continuous passive knee movement after total knee replacement surgery in 113 individuals. Active exercise led to higher proprioceptive acuity (*p* < 0.05), earlier ability to perform the straight leg raise test (*p* = 0.001), and improved performance in the Timed Up and Go Test (TUG) (*p* = 0.028), sit-to-stand (*p* = 0.05) and stair climbing (*p* = 0.038). However, only proprioceptive acuity remained significantly higher in the active group at 3-month follow-up. Lastly, Minshull et al. (30) found that passive stretching was equally effective as proprioceptive neuromuscular facilitation (PNF) in 18 healthy volunteers. Both interventions improved passive hip flexibility by 19.3% (*p* < 0.01) but had no effects on proprioceptive performance as measured by force reproduction error and active JPSE.

### Somatosensory Stimulation Training

Somatosensory stimulation was used as an intervention to improve proprioceptive and motor performance in ten studies. Somatosensory stimulation training that led to significant improvements in proprioceptive performance included Kinesio taping (62, 69), whole-body vibration (63), foam rolling of the lower extremity (65), and electrical stimulation of the hand (70). Five of the ten studies in this category reported significant improvements in proprioceptive performance, measured by JPSE, and seven studies reported significant improvements in motor performance (Figure 4). Mean treatment effects were 40% (range 5–78%) for proprioceptive performance and 25% (range 3–39%) for motor performance. Of the ten studies using somatosensory stimulation, Cho et al. (69) found the largest improvement in proprioceptive function. In this study in individuals with knee osteoarthritis, a single application of Kinesio Tape led to a 78% decrease in ankle JPSE at 45° plantarflexion (Pre-intervention M: 14.50° SD: 3.50°, Post-intervention M: 3.2° SD: 1.37°), while there was no change in JPSE in the control group which received a placebo tape application.

In all three studies that applied whole-body vibration, motor performance improvements were shown after the intervention in adults after anterior cruciate ligament (ACL) reconstruction (61), healthy elderly adults (64), and children with cerebral palsy (63). However, only Ko et al. (63) also showed improvements in proprioceptive performance. The authors assessed the effects of whole-body vibration in combination with standard physical therapy (*N* = 12) compared to physical therapy alone (*N* = 12). Participants received 9 min of whole-body vibration in addition to their 30-min therapy session, twice a week for 3 weeks. Children in the experimental group improved mean ankle proprioception by 54% and mean gait-related measures such as speed (23%), step length (25%), and step width (29%). Whole-body vibration led to significantly improved gait speed and step width when compared to the control group. The six studies that used Kinesio taping, massage, or foam rolling showed mixed results. In three studies, the interventions led to improved proprioceptive and motor performance measures (62, 65, 69), while three other studies showed no changes in either of the outcome measures (66–68).

There is initial evidence that somatosensory stimulation training is more effective in clinical than in non-clinical populations. The two studies assessing the effect of somatosensory stimulation in neurological populations (cerebral palsy, stroke) (63, 70) and two of three studies in orthopedic populations showed positive effects of somatosensory stimulation on proprioceptive function, measured by JPSE. On the other hand, only one of the five studies in healthy populations using somatosensory stimulation reported positive effects on proprioception.

Intervention duration ranged from a single application to 8 weeks. Interestingly, the two longest interventions of 8 weeks with two weekly sessions of whole-body vibration in people after ACL reconstruction (61) and in healthy adults (64) did not lead to significant improvements in proprioceptive acuity as measured by JPSE.

### Combined/Multiple System Training

Six studies applied combined or multiple systems interventions to improve proprioceptive and motor performance, all in the lower extremity. Five of those six studies used a combination of somatosensory stimulation and active movement training (72, 73, 75–77) and one study used a combination of passive stretching and active robot-assisted movement of the lower limb (74). The study populations included people with orthopedic lower limb injuries and neurological populations with stroke or multiple sclerosis. Five of the studies reported significant improvements in proprioceptive and motor outcomes (Figure 4). Relative mean treatment effects were 47% (range 17–92%) for proprioceptive performance and 86% (25–152%) for motor performance. Proprioceptive measures included JPSE and joint position sense detection threshold, while motor measures included clinical rating scales such the American Orthopaedic Foot and Ankle Society Ankle-Hindfoot Score (75), postural stability measures including CoP displacement and sway area (76, 77), and joint-specific measures such as knee-to-wall distance (73) and ankle passive ROM in dorsiflexion (74).

The combination of transcutaneous electrical nerve stimulation of calf muscles and active movement training implemented by Alahmari et al. (73) led to the largest improvement in proprioceptive function in individuals after an ankle sprain. The researchers assessed the effects of 3 weeks of combined somatosensory stimulation and active movement (*N* = 20), compared to active movement alone (*N* = 20) and no intervention (*N* = 20). Combined systems training led to an improvement in mean ankle JPSE of 92% (Pre-intervention: 2.5° SD 1.7°, Post-intervention M: 0.2° SD: 0.5°), while the other two groups showed no improvement. Smaller ankle JPSE indicates superior ankle proprioceptive function, which is essential for postural control and balance during standing and walking (86–88). The combined systems group was also superior to both the active movement training and control groups in post-intervention motor outcomes.

### Mind-Body Exercises

Mind-body awareness exercises such as meditation, Yoga, Tai Chi and Qigong have received increasing scientific attention in recent years and have shown to have positive effects on motor performance, depression and quality of life (89), as well as cognitive function (90). Considering the positive influence of such interventions on motor performance, researchers have also addressed the question whether mind-body exercises may positively affect proprioceptive performance. Five studies were included in this review assessed the effects of mind-body exercises using Tai Chi (12, 24), Yoga (41), walking meditation (78), and Yoga meditation (79). Study populations included individuals with ACL injury (12), Parkinson's disease (79), Type II Diabetes (24), and older adults (41, 78). All studies showed improvements in motor outcomes, and three studies (12, 24, 78) reported gains in proprioceptive acuity as measured by JPSE. Mind-body exercises were shown to be effective in improving proprioceptive and motor performance in a variety of populations, with improvements in JPSE ranging from 43 to 58%. Intervention duration ranged from 6 to 24 weeks, and dosage between 12 and 72 h. Empirical evidence on optimal dosage is inconclusive. Previous work suggested that a longer mind-body exercise intervention duration may be crucial in improving cognitive function and motor performance (89, 90). However, two mind-body intervention studies examining older adults (41) and people with Parkinson's disease (79) did not find gains in proprioceptive performance (dosage: 13.5 and 18 h over 6 and 12 weeks, respectively). Another study by Chatutain et al. (78) using a shorter intervention of 8 weeks with a total of 12 h of training did show improvements in both proprioceptive and motor outcomes. Their study trained 29 older adults in mindfulness using a walking meditation practice and compared proprioceptive and motor performance to a control group. Post-intervention, the intervention group showed significant improvements in active JPSE (43% mean reduction in angular error) and motor performance (34.1% increase in Functional Reach Test distance). For the control group, changes in proprioceptive and motor performance were not significantly different from baseline.

### Summary

Using relative improvement as a metric shows that across a wide range of training studies that reported statistically significant gains, proprioceptive performance improved on average by 46% and motor performance by 45%. Balance training and/or active movement interventions were used by a majority of studies (50 out of 70). Approximately 86% (43/50) of the studies showed improvement in at least one proprioceptive or motor measure, with 64% (32/50) of studies reporting gains in both proprioceptive and motor function.

The effects of passive movement training on proprioceptive performance are mixed and may depend on the population. Individuals who are able to perform active movements appear to benefit more from active training when one considers markers of proprioceptive and motor function. Nevertheless, passive movement interventions were shown to be equally effective in improving specific motor functions, such as passive joint flexibility. Similarly, results of somatosensory stimulation training to improve proprioception were mixed. Data from this review suggest that effects depend on the study population, such that neurological populations may benefit most consistently from such interventions. Accordingly, combined systems training, such as somatosensory stimulation in combination with active movement, appears to effectively improve proprioception, measured by JPSE and joint position detection threshold, and motor function in orthopedic and neurological populations. No studies using multiple systems training in non-clinical populations were included in this review. Mind-body exercises such as meditation, Tai Chi and Yoga were used to improve proprioceptive and motor performance in a variety of populations. While all studies showed improvements in motor performance, the study results indicate that longer intervention duration and dosage may be required lead to changes in proprioceptive performance.

### Classification of Proprioceptive Training by Study Population

**Figure 5:**
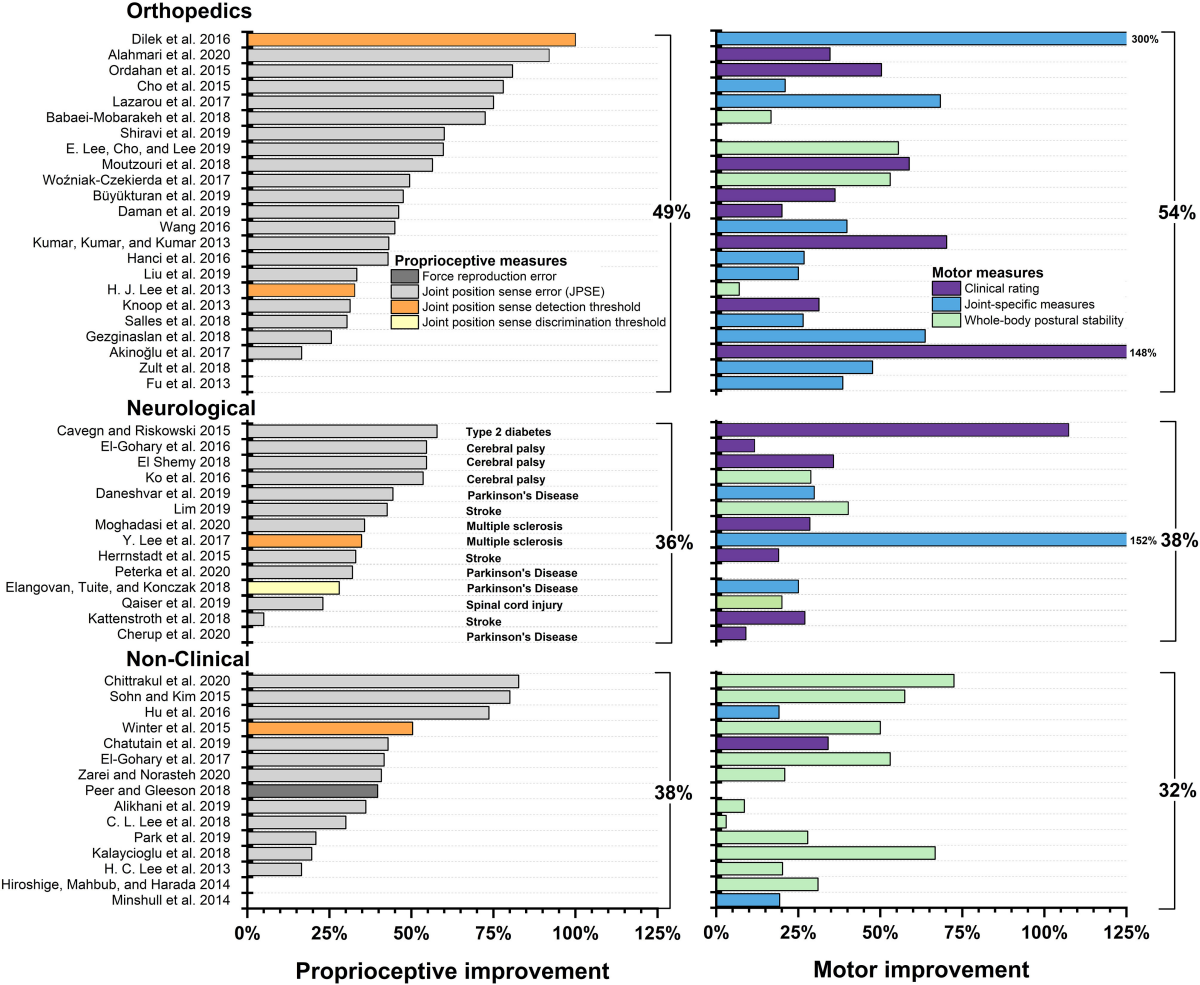
Treatment effects by population type. Studies not reporting exact data are not pictured and were not included in calculating the mean. For studies that applied multiple proprioceptive or motor measures, only the proprioceptive and motor measure with the largest percentage improvement between pre- and post-intervention measures is shown.

Interventions to improve proprioception and motor function have been used in a wide variety of populations. We categorized studies into three subgroups, according to the study population assessed. These subgroups were orthopedic, neurological and non-clinical populations. Figure 5 displays treatment effects of proprioceptive and motor measures. In addition, a small number of studies assessed the effect of training on proprioceptive and motor performance in populations affected by hypermobility, head and neck injuries, Diabetes mellitus, as well as deaf individuals.

### Orthopedic Populations

Because muscles, tendons, and ligaments contain proprioceptors, orthopedic injuries affecting these tissues are known to disrupt or alter proprioceptive signals, which has negative effects on motor function (91–94). Orthopedic injuries in studies in the present review included lower extremity injuries, such as ACL injuries, hip and knee joint arthroplasty, knee joint osteoarthritis, foot and ankle injuries, as well as joint hypermobility, neck and head, and upper extremity injuries. A total of 29 total studies measured proprioception and motor performance in people with orthopedic injuries. Twenty-three of these studies demonstrated post-intervention improvements in proprioceptive (range: 17–92%, M: 50%) or motor performance (range: 7–300%, M: 56%) (Figure 5). Effect sizes ranged from small to large for proprioceptive and motor measures (Tables 2, 3).

### Lower Extremity Injuries

Sixteen studies assessed the effect of proprioceptive training in individuals with hip and knee injuries, including ACL injury and knee joint osteoarthritis (45–47, 69), as well as hip (59) or knee arthroplasty (57–59, 77). Only three of these studies did not show post-intervention improvement in proprioceptive performance (15, 59, 61) and one study did not report post-intervention motor improvement (61). Interventions that led to significant post-test improvements included active multi-joint movements such as additional knee rehabilitation programs (13, 14), Tai Chi (12), neuromuscular knee joint facilitation (16), active heel-slide exercises (57), active training interventions targeting balance, agility, stability and/or strength (45–47, 58), as well as somatosensory stimulation in the form of Kinesio taping (62, 69, 77) and the application of radiofrequency shrinkage treatment on the ACL (72). Notably, all three studies that used Kinesio Tape (62, 69, 77) demonstrated proprioceptive benefits (relative change between 33 and 78%), and motor performance improvements (range 21–53%).

Six studies assessed the effects of proprioceptive training in individuals with ankle sprain (17, 73), ankle instability (20–22), or plantar fasciitis (75). Their mean improvement in ankle JPSE was 48% (range 17–92%) at post intervention. Mean motor scores were improved by 57% (range 7–148%). Interventions were predominantly active, including PNF (17) combined with transcutaneous electrical nerve stimulation of the calf (73), short foot exercises (20), strength training (22), and balance training combined with the use of ankle orthotics (21), but also included somatosensory stimulation (75). The largest proprioceptive performance improvement was shown by Alahmari et al. (73), who assessed the effects of a 5-week intervention that combined PNF and transcutaneous electrical nerve stimulation in 20 individuals with ankle sprain, compared to PNF alone (*N* = 20) and a control group (*N* = 20). Ankle JPSE in the experimental group improved by 92% (Pre-intervention: M: 2.5° SD: 1.7°, Post-intervention: M: 0.2° SD: 0.5°). The largest motor performance improvement was shown by Akinoglu et al. (75), who assessed the effects of radial extracorporeal shock wave therapy (*N* = 18) compared to ultrasound (*N* = 18) and a control condition (*N* = 18) in individuals with plantar fasciitis. The experimental group improved their American Orthopedic Foot and Ankle Association score by 148% (Pre-intervention: M: 30.11 SD: 12.49, Post-intervention: M: 74.72 SD: 13.55).

### Upper Extremity Injury

Four studies assessed the effect of proprioceptive training in individuals with upper extremity injuries including impingement syndrome or tennis elbow (54), infraspinatus muscle atrophy (44), subacromial impingement syndrome (55), and subacromial pain syndrome (56). Three of these studies demonstrated post-intervention improvements in proprioceptive and motor function. All studies used interventions involving active movement exercises at the shoulder or wrist. The one study that did not show post-intervention improvements (56) implemented a single session (total practice time: 36 s) of flexible bar exercises in individuals with subacromial pain (*N* = 50), compared to unaffected controls (*N* = 50). In contrast, the interventions in the three other studies lasted for 6–8 weeks. It is likely that the bout of exercise applied by Boarati et al. (56) was too short to elicit post-intervention differences.

### Neurological Populations

Sixteen studies assessed the effects of interventions on proprioception and motor performance in individuals affected by neurological disorders, such as cerebral palsy (CP), Parkinson's disease (PD), stroke and spinal cord injury, multiple sclerosis (MS), and diabetes mellitus. Effect sizes ranged from medium to large for both proprioceptive and motor measures (Tables 2, 3). Across studies, mean post-intervention proprioceptive outcome measures improved by 40% (range 5–58%). Mean post-intervention motor performance improved by 32% (range 9–107%), measured by various balance, gait, strength, and ROM tests. Measurements of lower extremity proprioception demonstrated the largest improvements in proprioception (35–58%, M: 46%). Of the nine studies assessing lower extremity proprioception, seven utilized whole-body training interventions [(12, 14, 37, 42, 50, 73, 95); Table 1] whereas the other two trained only the lower limb or ankle. Mean upper extremity proprioceptive measurements improved by 25% (range 5–33%).

### Cerebral Palsy

Cerebral Palsy (CP) is a congenital, neurological disorder caused by abnormal brain development that negatively affects sensorimotor function (96). Four studies assessed the effects of proprioceptive training in individuals with CP (18, 26, 63, 71). All four studies demonstrated post-intervention improvements in proprioceptive and motor function. Intervention types included somatosensory discrimination training, somatosensory stimulation and active/balance training. One study assessed somatosensory discrimination training of the hand (71), while the other three studies applied whole body training in addition to standard care. Whole-body interventions included somatosensory stimulation in the form of vibration (63), as well as Biodex balance training (19), and walking on a treadmill with eyes open or closed (18). In the three studies that applied whole-body training, proprioceptive performance was assessing JPSE in the lower extremities. These three studies exhibited some of the greatest improvements in proprioceptive performance within the neurological populations assessed in this review. Relative reduction in JPSE ranged from 54 to 55% at the knee (18, 19) and ankle (63). Relative improvements in gait and balance improved by 12 to 29% as measured by decreased step width (Pre-intervention: M: 15.83 cm SD: 5.89 cm, Post-intervention: M: 11.27 SD: 5.42 cm) (26), decreased modified TUG duration (Pre-intervention: M: 21.46 s, Post-intervention: M: 13.80 s) (18), and improved pediatric Berg Balance Scale score (Pre-intervention: M: 35.91 points SD: 1.74 points, Post-intervention: M: 40.04 points SD: 2.17 points) (63) (Figure 5).

### Parkinson's Disease

Parkinson's disease (PD) is a neurodegenerative disease leading to impaired motor function (e.g., bradykinesia, rigidity, tremor) and is associated with proprioceptive dysfunction (97). Four studies aimed to improve proprioceptive and motor performance in PD (4, 50, 51, 79). Two of the studies showed post-intervention improvement in both proprioceptive and motor measures (4, 50), and one study each found post-intervention improvement in either proprioceptive (51) or motor outcomes (79). Mean proprioceptive performance improved by 35% (range 28–44%), while mean motor performance improved by 21% (range 9–30%).

In three of four studies, active movement interventions were applied, including whole-body training (50, 51) and robot-aided training of the wrist joint (4). Whole-body exercise interventions included training on a trampoline or treadmill (50) and maximal amplitude movements and stretching (51). The wrist training incorporated playing a virtual reality game in which participants moved a ball to a target position on the screen (4). One study applied yoga meditation (79). Study duration varied from a single application to 12 weeks. Notably, the longest training intervention of 12 weeks of yoga meditation did not lead to proprioceptive performance improvements (79), while the single session of robot-aided wrist exercise (mean duration: 33 min) significantly improved both proprioceptive and motor performance (28% mean reduction in wrist joint position sense discrimination thresholds and 59% mean decrease in cumulative spatial motor error) (4).

### Stroke and Spinal Cord Injury

Stroke leads to brain tissue damage after reduced blood supply to the brain. Insults affecting sensorimotor cortex and/or its efferent projections impair proprioceptive and motor function. Partial or complete severing of the spinal cord also induces somatosensory motor impairment to varying degrees. Four studies measured the influence of training in people with subacute (70, 76) and chronic stroke (52, 53), all of which demonstrated improvements in proprioceptive and motor performance. Interventions included active movement such as matching tasks of the arm (52) and a robot-aided gamified finger exercise (53), as well as somatosensory stimulation which included repetitive sensory stimulation of the hand (70) and the combination of balance training and transcutaneous electrical nerve stimulation (76). One study measured the influence of proprioceptive training on proprioceptive and motor performance in 15 individuals with a spinal cord injury (60). During the intervention, participants' heels were passively moved to various positions, upon which the participant identified where the heel was in relation to the reference position. Knowledge of results via visual feedback was provided after each trial. The training was conducted over 12 h on multiple days. As a result, mean knee JPSE improved by 23% (Pre-intervention: M: 5.22° SD: 4.63°, Post-intervention: M: 4.03° SD: 3.05°) and precision stepping error improved by 20% (Pre-intervention: M: 18.69 mm SD: 8.76 mm, Post-intervention: M: 14.91 mm, SD: 7.80 mm).

Overall, mean proprioceptive performance improvement was 30% (range 5–43%). Training duration ranged from 2 days to 8 weeks, but four of the five studies implemented interventions of 3 weeks or less. It is noteworthy two of the studies implemented electrical stimulation as the intervention (70, 76), whereas no other neurological population applied this treatment type. Further, more general training interventions were longer in duration than more specific interventions. That is, more time was spent during exercise movements of the leg (40 h) than during electrical stimulation of the hand (7.5 h) or force-feedback matching movements of the arm (3 h).

### Multiple Sclerosis

Multiple Sclerosis (MS) is a progressive autoimmune disease of the CNS that leads to sensory and somatosensory impairments, including mobility restriction (98). Two studies utilized an intervention to influence proprioceptive and motor performance in individuals with MS (48, 74). Both studies demonstrated post-intervention improvement in lower extremity proprioceptive and motor measures and both studies used active movement interventions. The two studies demonstrated similar proprioceptive improvements in the lower extremity and implemented an intervention of similar duration (6–8 weeks with 12–13.5 total practice hours). Lee et al. (74) showed improvements in mean ankle joint position sense detection threshold of 35% (Pre-intervention: M: 3.82° SD: 2.52°, Post-intervention: M: 2.49° SD: 0.50°), while Moghadasi et al. (48) found a 36% improvement in knee JPSE (Pre-intervention: M: 5.15° SD: 2.26°, Post-intervention: M: 3.31° SD: 1.33°).

### Non-clinical Populations

Non-clinical populations were subcategorized into *Athletes and younger adult non-athletes* and *elderly adults/fall prevention*. Twenty-four studies assessed non-clinical populations, of which only 11 demonstrated post-intervention improvements in proprioceptive performance. Motor improvements were shown in 16 studies. The study by Hiroshige et al. (64) measured both young and elderly healthy individuals. This study is evaluated in both the non-athlete and elderly/fall prevention sections. Overall, participants improved proprioception by 47% (range 20–83%) and motor performance by 36% (range 4–72%). Effect sizes ranged from small to large for proprioceptive and motor measures (Tables 2, 3).

### Athletes and Young Adults

Only three out of the 16 studies targeting young adults or young athletes demonstrated improvements in proprioceptive and motor performance while the other three studies found no difference in either proprioceptive or motor measures (33, 68, 80). The interventions leading to performance improvements included active multi-joint movements such as plyometric training for badminton players (25), a core strengthening program for dancers (28) as well as an ankle strength, position sense and balance training program for speed skaters (36). All of those studies aimed to improve proprioceptive performance in the lower extremity with interventions ranging from 6 to 12 weeks.

Ten studies assessed the effects of proprioceptive training on proprioceptive and motor performance in young healthy non-athletes (Table 1). Five of these studies demonstrated proprioceptive improvements, while six studies reported motor improvements. Proprioceptive performance improved on average by 41% (range 21–74%) in 5 studies and mean motor performance improved by 25% (range 4–53%). Active and balance interventions were used predominately; somatosensory stimulation was used in one study. Active interventions included neuromuscular wrist joint facilitation (27), balance training (26, 32) and proprioceptive exercises using augmented reality (31), while somatosensory stimulation was applied in the form of non-vibration foam rolling (31). The total training time and training duration were short in comparison to interventions in other populations. Total training time ranged from 6 min to 4 h, while training duration ranged from a single application to 8 weeks with three studies implementing single-application interventions and two studies implementing interventions on three separate days. It is possible that training was under-dosed in the populations of healthy non-athletes, as three of the five studies that did not lead to proprioceptive improvements were among the studies of shortest intervention duration.

### Elderly/Fall Prevention

Of the seven studies targeting elderly individuals, three studies demonstrated improvements in post-intervention proprioceptive performance (range 43–83%) and five in motor measures (range 34–72%). The interventions that led to proprioceptive improvement included active movement such as whole-body exercise in the form of strength, reaction time, and balance training. Intervention lengths ranged from 6 weeks to 12 months. Notably, participants in the study with the longest training duration (39) showed only minimal improvements in gait speed but declined balance and proprioceptive performance at post-intervention.

### Summary

Proprioceptive interventions were effective in improving proprioceptive and motor performance in orthopedic, neurological, and in non-clinical populations (Figure 5). In orthopedic populations, 23 out of 29 studies (79%) yielded statistically significant gains in both proprioceptive and motor performance measures. In a large majority of studies, active exercise such as Tai Chi or strength training was applied. Of the studies that implemented an exercise intervention, just over half targeted a single body part (i.e., knee, ankle). Somatosensory stimulation was also shown to be effective, particularly Kinesio taping. Intervention duration varied greatly between a single bout of exercise or somatosensory stimulation to exercise regimen lasting up to 36 weeks. There was no clear pattern that might indicate an optimal training dosage.

In individuals with neurological disorders, proprioceptive training interventions generally improved both proprioceptive and motor performance. Fourteen of the 16 studies (88%) demonstrated improvements in proprioceptive performance and 15 of 16 studies (94%) showed improved motor function. Interventions targeting the lower extremity were associated with larger post-intervention improvements than those targeting the upper extremity.

Interventions in non-clinical populations were the least effective in this review. Of a total of 24 studies in athletes and non-athletes, less than half (11/24) led to improvements in proprioceptive performance. The three studies demonstrating the greatest improvements in proprioception were studies in elderly/fall prevention and young non-athlete populations. In the young individuals, exercise was the intervention used in all but one study that demonstrated an improvement in proprioceptive performance. All but one study (27) measured proprioception in the lower extremities. That is, of the 11 studies documenting improvements in proprioceptive function, 10 measured knee or ankle joint proprioception. The largest improvements in motor function were the result of strength training and balance exercises.

## Discussion

This review aimed to provide the current state of research on the effects of a proprioception-focused sensorimotor training on proprioceptive and motor function. Specifically, we (a) documented the types of interventions that have been applied to improve proprioception and motor performance, (b) highlighted what outcome measures were used to quantify the effects on proprioceptive and motor performance, and (c) examined the usefulness of proprioceptive training approaches as a rehabilitation tool to improve motor function and performance in clinical populations.

### Which Interventions Are Most Effective?

A fundamental question to answer is what type of interventions are most successful in improving proprioceptive and motor function. In addition, it would be important to know whether an intervention that specifically targets proprioceptive-motor function is superior to traditional forms of multimodal sensorimotor training. This knowledge could guide future training approaches in such diverse fields as athletic performance or physical rehabilitation. Our review of the recent empirical evidence allows for some general observations: First, when considering active movement/balance interventions, the reported gains in proprioceptive function were generally comparable to improvements in motor function. That is, those studies showing large proprioceptive improvements also showed large improvements in motor performance. Second, for those interventions that targeted somatosensory function (e.g., passive movement, somatosensory discrimination training and somatosensory stimulation training) the post-intervention effects on proprioceptive function tended to be slightly higher than changes in motor performance. Such training predominantly targets proprioceptive function while indirectly improving motor function. Third, our analysis revealed that a large variety of training types positively influenced proprioceptive and motor performance. There is no single intervention that stands out as being most successful. Proprioceptive training was effective in improving proprioceptive performance in 71% of the studies (50/70) and improved motor performance in 81% of those reports (57/70). In general, the magnitude of gain for proprioceptive outcome measures was 20% or more in 43 out of 50 studies and motor outcome measures improved by 20% or more in 41 out of 57 studies. A vast majority of studies (55 out of 70) used active movement alone or a combination of active movement and somatosensory stimulation to improve proprioceptive and motor performance.

The effectiveness of training on proprioceptive and motor performance is further substantiated by the analysis of the effect sizes of within- or between-group differences of the 40 studies that provided sufficient information for calculation. Effect sizes for proprioceptive outcomes were medium or large (*d* ≥ 0.5, η^2^ ≥ 0.06) (Tables 2, 3) in 32 out of 36 studies. Similarly, the effect sizes for the corresponding motor outcome measures were medium or large in 38 out of 40 studies. Although there was no apparent pattern of effect sizes regarding intervention types, it is noteworthy that interventions in neurological populations all led to medium or large effect sizes. However, effect sizes for within-group comparisons in studies applying somatosensory stimulation or discrimination training were small or medium (*d* ≤ 0.5, η^2^ ≤ 0.06), indicating that somatosensory training alone may be less effective in improving proprioceptive and motor performance than sensorimotor training.

With respect to the optimal training dosage or intensity, no consistent overall pattern emerged that could guide future research, clinical trials, and practice. However, for balance interventions, longer durations of 6 weeks or more appeared to be more successful than shorter interventions of only 3 weeks (32, 59). In contrast, somatosensory stimulation led to rapid changes in proprioceptive and motor performance even within a single session. Moreover, there is initial evidence that interventions targeting a single joint or body segment required shorter intervention duration and dosage and led to more frequent and larger improvements when compared to whole-body interventions of similar dosage.

Finally, it would be desirable to know how training dosage influences the time that any proprioceptive-motor gains are retained. Unfortunately, a majority of studies did not perform follow-up assessments to test for retention of training gains. Those studies that did perform follow-up assessments varied greatly in their retention interval, with intervals ranging between 1 day (62) and 26 weeks (46). Thus, at present there is no clear evidence from which to draw firm conclusions on how long gains after proprioceptive and motor learning persist. Yet, this information is vitally important to inform future training protocols for rehabilitation interventions in clinical populations.

### Which Proprioceptive Outcome Measures Are Most Sensitive to Detect Gains?

The studies reviewed here used a variety of heterogeneous outcome measures, which makes it difficult to directly compare results across studies. The majority of the studies reported some form of *joint position sense error* as an objective measure of proprioceptive acuity (the ability to discriminate between different stimuli) (99). However, one needs to recognize that a JPSE can be computed under a range of different experimental paradigms. Typical joint position matching tasks either use ipsilateral or contralateral matching. During ipsilateral matching the same limb is moved consecutively to two distinct positions and JPSE marks the difference between the two assumed positions. In contralateral matching task, one limb is moved (e.g., the right forearm) and the assumed joint position is matched by the contralateral, homologous limb (e.g., the left forearm). All studies reporting JPSE in this review used an ipsilateral joint position matching paradigm. Ipsilateral matching tasks rely on working memory as the previous position needs to be remembered in order to be able to match. In contrast, contralateral testing eliminates the memory issue as both joint positions are experienced simultaneously, but it introduces potential bias from limb preference (e.g., handedness or footedness) and it relies on intact neural processing across the two brain hemispheres (83, 84). Work by Goble et al. (83) and Elangovan et al. (100) have shown that in healthy adults, ipsilateral joint position matching tends to lead to a smaller JPSE than contralateral matching. In addition, it is important to consider, whether a JPSE was obtained by passively displacing limbs or whether the examinee actively moved the limb to match a joint position. One needs to recognize that an active movement-based JPSE is a composite measure of both proprioceptive and motor function. This active movement-based approach becomes problematic in clinical populations with known impairments of motor function, as it becomes difficult to discern to what extent a JPSE is a measure of motor dysfunction or the result of impaired proprioception. When testing proprioceptive function in people with compromised motor control, it is therefore advisable to employ passive movement-based forms of testing. Furthermore, joint position matching task performance is influenced by several factors, including how long a limb position is presented, limb preference and participant age (83).

As an alternative to JPSE, psychophysical threshold hunting techniques can be employed. These methods represent an established gold standard for assessing proprioceptive senses and yield detection or discrimination thresholds that are comparable across studies (Figure 2). However, they typically require specialized equipment, and the devices are restricted to assess single-joint function. Importantly, proprioceptive thresholds derived under conditions where a joint is passively rotated represent the closest estimation of “pure” proprioception as influences input from other sensory modalities and higher order neural processes of sensory and sensorimotor integration can be controlled.

### Which Populations Benefitted Most From Proprioceptive Interventions?

Our analysis showed that a wide variety of populations may benefit from a proprioceptive-focused intervention. Such training regimens applied to clinical populations led to marked positive changes in proprioceptive and motor function more frequently than those in non-clinical populations. It is noteworthy that the largest improvements in non-clinical populations were not in athletes but in elderly adult/fall prevention populations. Reasons for this may include larger potential for improvement in untrained healthy elderly adults compared to athletes, as well as the fact that interventions were generally shorter in the athlete populations and may have been insufficient to lead to significant changes. It has previously been stated that somatosensory rehabilitation is crucial in neurorehabilitation as somatosensory loss is associated with poorer motor recovery (101) and impaired motor learning (102). Results from this review demonstrate that both orthopedic and neurological populations improve proprioceptive and motor performance following interventions targeting proprioception, underlining the importance of targeting somatosensation in addition to motor performance in rehabilitation programs.

### Challenges in Evaluating Existing Literature

While some general conclusions can be drawn, open questions and challenges remain. First and foremost, the terminology to describe the tested sensory modalities is inconsistent. For example, some studies claimed to examine joint motion sense detection thresholds (36, 41, 44, 46, 55, 74, 79). All of those studies, passive joint angular displacement was performed at a single, constant, slow speed (range from 0.3 to 1.5 deg/s) and participants were instructed to indicate once they became aware of their limb segment being moved. Subsequently, the time or angular displacement were measured. For the purpose of this review, this proprioceptive measure was categorized as joint *position* sense detection threshold instead of *motion* sense detection threshold, as stated in the studies. The following reasoning underlies this decision: Measuring the time or angular displacement at a constant velocity provides a measure of joint position sense detection threshold, whereas velocity is a measure of motion sense detection threshold. In order to detect joint motion sense detection thresholds, the joint needs to be moved at a *variety* of angular velocities to identify the velocity threshold at which motion can be detected in the respective joint (103). Second, the current body of literature presents with a lack of consensus on what constitutes *proprioceptive training*. In a strict sense, an intervention that allows for multimodal sensory input during a motor training and then measures gains in proprioceptive function, is not proprioceptive training. As we previously put forward “*proprioceptive training* is an intervention that targets the improvement of proprioceptive function, focusing on the use of somatosensory signals such as proprioceptive or tactile afferents in the absence of information from other modalities such as vision. Its ultimate goal is to improve or restore sensory and/or sensorimotor function” (1). Considering this operational definition, most interventions included in this review would not be considered pure forms of proprioceptive training because they did not restrict information from other sensory modalities such as vision. While such multimodal sensorimotor training may be desired, it makes it difficult to discern if reported gains in motor function were driven by enhanced proprioceptive processing or by optimizing other neural processes such as multimodal sensory integration, sensory-motor integration, or improved motor execution.

Studies included in this review did not address fundamental questions regarding central mechanisms for the rehabilitation of proprioceptive and motor deficits, such as after ACL injury, despite known associations between central processing and changes in performance post-injury (104, 105). Better understanding of the relationship between neurophysiological changes, proprioceptive impairment and improvement is necessary to adequately address these issues. Despite the lack of evidence for changes associated with the central nervous system in the present study, a body of empirical research has shown that changes in sensorimotor function are associated with central reorganization (6, 104, 106–108). Further, sensorimotor intervention has been shown to improve and restore neuro-cognitive functions in recent neuroimaging research. In particular, sensorimotor intervention has been used to re-establish sensorimotor strategies in body representation (109). These neuro-cognitive functions are associated with activation of specific neural networks (110), as well as cortico-spinal pathways (111). The present systematic review substantiates the understanding of behavioral aspects of the effects of training aimed at improving proprioceptive and motor function. This underlines and extends current knowledge of the relationship between behavioral and neuroscientific evidence on the effects of proprioceptive training.

### Resume and Recommendations for Future Studies

The last decade has seen an increased effort in gaining a more complete understanding of the close link between proprioceptive and motor function, and a vast number of studies that apply forms of proprioceptive-focused interventions to improve motor outcomes. It is noteworthy that the body of literature using distinct proprioceptive measures has increased substantially since our previous systematic review which summarized work up to 2013 (1). There is now convincing empirical evidence that approaches such as active movement and balance interventions can induce large gains in both proprioceptive and motor function. Further, there is evidence that interventions aimed at improving somatosensory function do not only improve proprioception but also motor function, supporting the notion that somatosensory training induces cortical reorganization (1). Behavioral changes induced by proprioceptive training fit with current knowledge of neurophysiological changes associated with sensorimotor interventions (109), providing new insights on possible benefits of proprioceptive training and underlining how multifaceted the effects of proprioceptive training are.

A major, consistent problem is the plethora of reported outcome measures that are not directly comparable, which then does not allow for a comparison between studies. This use of outcome measures severely impacts our understanding of “what works” and the limits the scientific impact of the studies. In order to address this issue, it is paramount that future studies apply a consistent terminology and use established objective motor and proprioceptive outcome measures that are sensitive and reliable. Researchers need to be aware that the type of employed assessment technique using either active or passive motion, focusing on a single joint or representing a multi-joint composite measure, will constrain the interpretation of their results and will affect their conclusions.

Finally, our current knowledge on how well the reported proprioceptive and motor gains are retained after practice is rather incomplete. Many studies failed to examine retention at all. Yet, solid empirical evidence on observable short- and long-term retention after proprioceptive training is imperative to understand the underlying mechanisms of neural plasticity, and to delineate the neuroanatomical regions as well as the neurophysiological processes of proprioceptive-motor learning. There is a need within the scientific community to harmonize outcome measures and to apply proven outcome measures that are part of an accepted toolbox. This would allow future studies to select appropriate motor and proprioceptive measures for the clinical or non-clinical population under study that will be comparable to other research-specific combinations of in order to increase comparability of results.

## Data Availability Statement

The original contributions presented in the study are included in the article/Supplementary Materials, further inquiries can be directed to the corresponding author/s.

## Author Contributions

LW, QH, and JS conducted the literature search and subsequent analyses, and wrote sections of the manuscript. All authors contributed to manuscript revision, read, and approved the submitted version.

## Funding

This research was supported by funds from the University of Minnesota Marty and Jack Rossmann Award to JK.

## Conflict of Interest

The authors declare that the research was conducted in the absence of any commercial or financial relationships that could be construed as a potential conflict of interest.

## Publisher's Note

All claims expressed in this article are solely those of the authors and do not necessarily represent those of their affiliated organizations, or those of the publisher, the editors and the reviewers. Any product that may be evaluated in this article, or claim that may be made by its manufacturer, is not guaranteed or endorsed by the publisher.
