# Supplementary material for: The Effectiveness of Proprioceptive Training for Improving Motor Performance and Motor Dysfunction: A Systematic Review
Source: Front Rehabil Sci. 2022 Apr 8;3:830166. doi: 10.3389/fresc.2022.830166 (PMC9397687; doi:10.3389/fresc.2022.830166)
Supplement: Supplementary file 1 [file Data_Sheet_1.pdf]

Supplementary Material

Appendix A: Search strategies

Searches in the databases were conducted on October 20, 2020 using the following strategies:

Medline (Ovid):

1) propriocept*.mp.

2) exp Proprioception/

3) exp Rehabilitation/ or rehabilitation.mp.

4) exp Physical Therapy Modalities/

5) proprioceptive training.mp.

6) training.mp.

7) 1 or 2

8) perceptual motor learning.mp.

9) motor processes.mp.

10) exp Kinesthesis/ or kinesthetic perception.mp.

11) Discrimination Learning/

12) (“form and shape perception”).mp.

13) exp Learning/ or learning.mp.

14) motor learning.mp.

15) exp Central Nervous System Diseases/ or CNS disorders.mp.

16) exp Musculoskeletal Diseases/ or musculoskeletal disorders.mp.

17) muscular disorders.mp.

18) 15 or 16 or 17

19) 9 or 18

20) 1 or 2 or 10 or 12

21) 5 or 8 or 11 or 14

22) 6 or 13 or 21

23) 19 and 20 and 21

24) limit 23 to (humans and yr="2013 -Current" and (chinese or english or german))

25) 20 and 21 26) limit 25 to (humans and yr="2013 -Current" and (chinese or english or german))

27) 1 or 2 or 5

28) 3 or 4

29) 15 or 16 or 17

30) 27 and 28 and 29

31) limit 30 to (humans and yr="2013 -Current" and (chinese or english or german))

32) exp Postural Balance/

33) 1 or 2 or 10 or 12 or 32

34) 21 and 33

35) neuromuscular training.mp.

36) 21 or 35

37) 33 and 36

CINAHL:

(MH proprioception+ OR propriocept*) AND (therapy or treatment or rehabilitation or intervention) AND (learning or training or exercise) NOT Meta-analysis or systematic review in Title or Abstract, limited 2013-current, limited research article & peer reviewed, limit to languages (Chinese, English, German)

SCOPUS:

TITLE-ABS-KEY (propriocept*) AND TITLE-ABS- KEY (human AND (controlled AND study) AND training) AND (LIMIT-TO (PUBYEAR, 2020) OR LIMIT-TO (PUBYEAR, 2019) OR LIMIT-TO (PUBYEAR, 2018) OR LIMIT-TO (PUBYEAR, 2017) OR LIMIT-TO (PUBYEAR, 2016) OR LIMIT-TO (PUBYEAR, 2015) OR LIMIT-TO (PUBYEAR, 2014) OR LIMIT-TO (PUBYEAR, 2013)) AND (LIMIT-TO (LANGUAGE, "English") OR LIMIT-TO (LANGUAGE, "German") OR LIMIT-TO (LANGUAGE, "Chinese"))

PsycInfo (Ovid)

1. Proprioception.mp. or exp Proprioception OR (Form and shape perception*).mp. OR (Kinesthetic Perception.mp. or exp Kinesthetic Perception) OR (discrimination/ or cognitive discrimination/ or perceptual discrimination/ or stimulus discrimination/ or discrimination learning/ or perception)

2. (Perceptual motor learning).mp. or exp (Perceptual Motor Learning) OR learning.mp. OR (Proprioceptive training).mp.

3. (Muscular disorders.mp. or exp Muscular Disorders) OR (Motor Processes).mp. or (exp Motor Processes) OR (exp Central Nervous System Disorders)/ or (CNS diseases).mp. OR (Musculoskeletal disorders.mp. or exp Musculoskeletal Disorders)

4. 1 AND 2 AND 3

5. limit 4 to (human and (chinese or english or german) and yr="2013 -Current")

Appendix B: Effect sizes for between-group comparisons showing Cohen's *d* and Hedge's *g*. For studies that applied multiple proprioceptive or motor measures, respectively, only the proprioceptive and motor measure with the largest improvement between pre- and post-intervention measures is shown.

**Study**	**Disease Entity**	**Outcome Measure**	**Sample Size**		**SD_{pooled}**	**Cohen's d**	**95 % CI**		**Hedge's g**	**95 % CI**	
			Control	Training			LL	UL		LL	UL
**Active movement/balance**											
**Alikhani et al., 2019**	Healthy adults	Active JPSE	10	12	1.497	1.576	0.717	2.795	1.516	0.556	2.476
		Lower Quadrant Y-Balance test			7.596	†1.438	†0.586	†2.618	†1.383	†0.443	†2.324
**Büyükturan et al., 2019**	ACL injury (partial)	Active JPSE	29	29	1.265	1.344	0.811	1.982	1.326	0.756	1.896
		International Knee Documentation Committee-2000			16.415	†0.256	†-0.262	†0.794	†0.252	†-0.264	†0.769
**Chittrakul et al., 2020**	Healthy elderly adults	JPSE	36	36	1.300	2.039	1.517	2.684	2.017	1.447	2.586
		CoP displacement			364.964	1.552	1.061	2.139	1.536	1.001	2.062
**Daman et al., 2019**	Hypermobility syndrome	Active JPSE	12	12	2.508	1.176	0.373	2.225	1.136	0.269	2.002
		SF-36 - Physical functioning			9.47	†1.197	†0.393	†2.251	†1.156	†0.287	†2.025
**Daneshvar et al., 2019**	Parkinson's Disease	JPSE	10	10	2.874	0.967	0.092	2.094	0.926	0.000	1.853
		Range of motion knee			12.855	†1.262	†0.382	†2.467	†1.208	†0.249	†2.249
**Eymir, Erduran, and Ünver, 2020**	Total knee replacement	Active JPSE (day of discharge)	55	58	2.709	0.997	0.620	1.412	0.990	0.599	1.381
		TUG (day of discharge)			26.74	0.430	0.061	0.816	0.427	0.054	0.800
**El Shemy, 2018**	Cerebral Palsy	Active JPSE	15	15	3.057	1.963	1.191	3.043	1.910	1.039	2.781
		modified TUG			2.876	1.878	1.114	2.938	1.827	0.968	2.686
**El-Gohary et al., 2016**	Healthy adults	Active JPSE	30	30	1.487	0.874	0.366	1.45	0.863	0.333	1.393
		Stability Index (AP direction)			1.523	1.773	1.224	2.453	1.750	1.152	2.347
**El-Gohary et al., 2017**	Cerebral Palsy	Passive JPSE	24	24	1.371	0.766	0.200	1.406	0.753	0.167	1.340
		Gross motor function (walking)			4.393	†0.824	†0.257	†1.471	†0.811	†0.221	1.400
		Active JPSE	12	12	1.051	3.331	2.980	5.022	3.215	1.979	4.451

**Hu et al., 2016**	Healthy adults	Writing time			6.912	1.071	0.270	2.095	1.034	0.178	1.889
**Kumar, Kumar, and Kumar, 2013**	Knee osteoarthritis	Active JPSE	22	22	1.961	1.117	0.518	1.833	1.100	0.461	1.732
		Western Ontario and McMaster Universities Osteoarthritis Index			2.971	1.027	0.431	1.731	1.008	0.379	1.637
**E. Lee, Cho, and Lee, 2019**	Chronic ankle instability	Active JPSE	15	15	N/A	*eta^2=0.14	N/A	N/A	N/A	N/A	N/A
		Stability Index			N/A	*eta^2=0.192	N/A	N/A	N/A	N/A	N/A
**Merom et al., 2016**	Healthy elderly adults	Active JPSE	147	275	N/A	not sig.	N/A	N/A	N/A	N/A	N/A
		Center of pressure displacement			165.154	†-0.279	†-0.482	†0.078	†0.278	†0.077	†0.479
**Salles et al., 2018**	Infraspinatus muscle atrophy	Active JPSE	18	18	0.544	2.595	1.822	3.697	2.537	1.651	3.424
		Shoulder external rotation peak torque			6.017	†1.612	†0.929	†2.505	†1.576	†0.824	†2.328
**Shiravi et al., 2019**	Neck pain	Active JPSE	44	43	0.523	5.736	4.894	6.85	5.686	4.735	6.636
		Isometric scapular upward rotation muscle strength			N/A	not sig.	N/A	N/A	N/A	N/A	N/A
**Sohn and Kim, 2015**	Healthy adults	Passive JPSE	6	6	N/A	not sig.	N/A	N/A	N/A	N/A	N/A
		Isokinetic knee extension muscle strength†			7.663	†2.505	†1.272	†4.945	†2.313	†0.801	†3.824
**Zarei and Norasteh, 2020**	Deaf	Active JPSE	10	10	N/A	N/A	N/A	N/A	N/A	N/A	N/A
		Single leg stance (eyes closed)			2.155	†1.949	†1.024	†3.367	†1.866	†0.802	†2.931
**Somatosensory stimulation Training**											
**Cho et al., 2015**	Knee osteoarthritis	Active JPSE	23	23	4.008	2.670	1.969	3.629	2.624	1.829	3.419
		Pain-free active range of motion			10.847	†1.872	†1.244	†2.684	†1.840	†1.146	†2.533
**Fu et al., 2013**	ACL reconstruction	Passive JPSE	20	19	N/A	not sig.	N/A	N/A	N/A	N/A	N/A
		Stability Index (AP direction)			1.843	0.873	0.245	1.608	0.856	0.198	1.513
**Naderi, Rezvani, and Degens, 2020**	Healthy adults	JPSE*	40	40	N/A	0.93	N/A	N/A	N/A	N/A	N/A
		Isokinetic knee extension muscle strength*			N/A	0.66	N/A	N/A	N/A	N/A	N/A
**Somatosensory Discrimination Training**											
**McLean et al., 2017**	Hemiplegic Cerebral Palsy	Active JPSE	10	8	N/A	N/A	N/A	N/A	N/A	N/A	N/A
		Goal Attainment Scaling*			N/A	0.79	N/A	N/A	N/A	N/A	N/A
**Combined/Multiple System Training**											
**Akinoğlu et al., 2017**	Plantar Fasciitis	Passive JPSE	18	18	1.916	0.819	0.166	1.581	0.801	0.121	1.481
		American Orthopedic Foot and Ankle Society Ankle-Hind foot Score			11.637	†1.308	†0.642	†2.145	†1.279	†0.559	†1.999
		Passive JPSE	20	20	0.985	2.234	1.533	3.189	2.189	1.400	2.979

**Alahmari et al., 2020**	Ankle Sprain	Knee-to-wall distance (cm)			1.020	†2.942	†2.164	†4.049	†2.883	†1.989	†3.778
**Lim, 2019**	Stroke	Active JPSE	15	15	2.743	†0.558	†0.016	†1.367	†0.543	†-0.187	†1.272
		Stability Index			0.78	†0.577	†0.141	†1.389	†0.561	†-0.169	†1.292
**Wei et al., 2013**	ACL relaxation	Passive JPSE	15	16	N/A	N/A	N/A	N/A	N/A	N/A	N/A
		Lysholm Knee Score			7.36	†0.666	†0.039	†1.475	†0.648	†-0.075	†1.372
**Meditation**											
**Chatutain et al., 2019**	Healthy elderly adults	Active JPSE	29	29	1.681	1.071	0.549	1.677	1.057	0.506	1.607
		Functional Reach Test			5.064	†2.113	†1.531	†2.857	†2.085	†1.442	†2.727
**Cherup et al., 2020**	Parkinson's Disease	Joint position sense detection threshold*	18	15	N/A	†0.72	†0.1	†1.4	N/A	N/A	N/A
		Tinetti Score*			3.141	0.77	0.1	1.5	N/A	N/A	N/A

Legend: †*Direction of effect size was converted that increment indicates improvement. *effect size reported in study (Cohen's d unless otherwise specified).* AP – anterior-posterior; JPSE – Joint position sense error; N/A – data not available; TUG – Timed Up and Go Test.
